# Evaluating the potential for respiratory metagenomics to improve treatment of secondary infection and detection of nosocomial transmission on expanded COVID-19 intensive care units

**DOI:** 10.1186/s13073-021-00991-y

**Published:** 2021-11-17

**Authors:** Themoula Charalampous, Adela Alcolea-Medina, Luke B. Snell, Tom G. S. Williams, Rahul Batra, Christopher Alder, Andrea Telatin, Luigi Camporota, Christopher I. S. Meadows, Duncan Wyncoll, Nicholas A. Barrett, Carolyn J. Hemsley, Lisa Bryan, William Newsholme, Sara E. Boyd, Anna Green, Ula Mahadeva, Amita Patel, Penelope R. Cliff, Andrew J. Page, Justin O’Grady, Jonathan D. Edgeworth

**Affiliations:** 1 Centre for Clinical Infection and Diagnostics Research, Department of Infectious Diseases, School of Immunology and Microbial Sciences, Kings College London, London, UK; 2 Infection Sciences, Viapath, St Thomas’ Hospital, London, UK; 3 Department of Infectious Diseases, Guy’s and St Thomas’ Hospital NHS Foundation Trust, London, UK; 4 Quadram Institute Bioscience, Norwich Research Park, Norwich, UK; 5 Critical Care Directorate, Guy’s and St Thomas’ Hospital NHS Foundation Trust, London, UK; 6 Department of Cellular Pathology, Guy’s and St Thomas’ NHS Foundation Trust, London, UK

## Abstract

**Background:**

Clinical metagenomics (CMg) has the potential to be translated from a research tool into routine service to improve antimicrobial treatment and infection control decisions. The SARS-CoV-2 pandemic provides added impetus to realise these benefits, given the increased risk of secondary infection and nosocomial transmission of multi-drug-resistant (MDR) pathogens linked with the expansion of critical care capacity.

**Methods:**

CMg using nanopore sequencing was evaluated in a proof-of-concept study on 43 respiratory samples from 34 intubated patients across seven intensive care units (ICUs) over a 9-week period during the first COVID-19 pandemic wave.

**Results:**

An 8-h CMg workflow was 92% sensitive (95% CI, 75–99%) and 82% specific (95% CI, 57–96%) for bacterial identification based on culture-positive and culture-negative samples, respectively. CMg sequencing reported the presence or absence of β-lactam-resistant genes carried by *Enterobacterales* that would modify the initial guideline-recommended antibiotics in every case. CMg was also 100% concordant with quantitative PCR for detecting *Aspergillus fumigatus* from 4 positive and 39 negative samples. Molecular typing using 24-h sequencing data identified an MDR-*K. pneumoniae* ST307 outbreak involving 4 patients and an MDR-*C. striatum* outbreak involving 14 patients across three ICUs.

**Conclusion:**

CMg testing provides accurate pathogen detection and antibiotic resistance prediction in a same-day laboratory workflow, with assembled genomes available the next day for genomic surveillance. The provision of this technology in a service setting could fundamentally change the multi-disciplinary team approach to managing ICU infections. The potential to improve the initial targeted treatment and rapidly detect unsuspected outbreaks of MDR-pathogens justifies further expedited clinical assessment of CMg.

**Supplementary Information:**

The online version contains supplementary material available at 10.1186/s13073-021-00991-y.

## Background

The intensive care unit (ICU) is a dynamic environment with frequent staff-patient contact for invasive monitoring, interventions and personal care that together introduce the risk of secondary or nosocomial infection [1]. Invasive ventilation can introduce organisms into the lungs causing ventilator-acquired pneumonia (VAP) which carries high attributable mortality and drives up to 70% of antimicrobial prescribing [2]. Patients with suspected VAP receive guideline-directed empiric antibiotics until culture results return, typically 2–4 days later [3]. Invasive pulmonary aspergillosis (IPA) is also increasingly recognised on ICU particularly with severe influenza [4] and after host immunosuppression, but culture lacks sensitivity and biomarker tests have low specificity and long turnaround times whilst gold standard histopathology is rarely used [5].

SARS-CoV-2 has put considerable strain on ICUs, due to the expansion of bed capacity with severely unwell patients, which has the potential to increase nosocomial infection, antimicrobial treatment and antimicrobial resistance (AMR). A high prevalence of Gram-negative bacteria (GNB) particularly *Klebsiella* spp. has been reported [6], and there are reports of secondary IPA [7]. COVID-19 patients also receive steroid therapy, which could exacerbate bacterial or fungal infections [8]. The COVID-19 pandemic therefore re-enforces the need for rapid comprehensive diagnostics to improve antimicrobial stewardship and help prevent emergence and transmission of multi-drug-resistant (MDR) organisms.

**Fig. 1:**
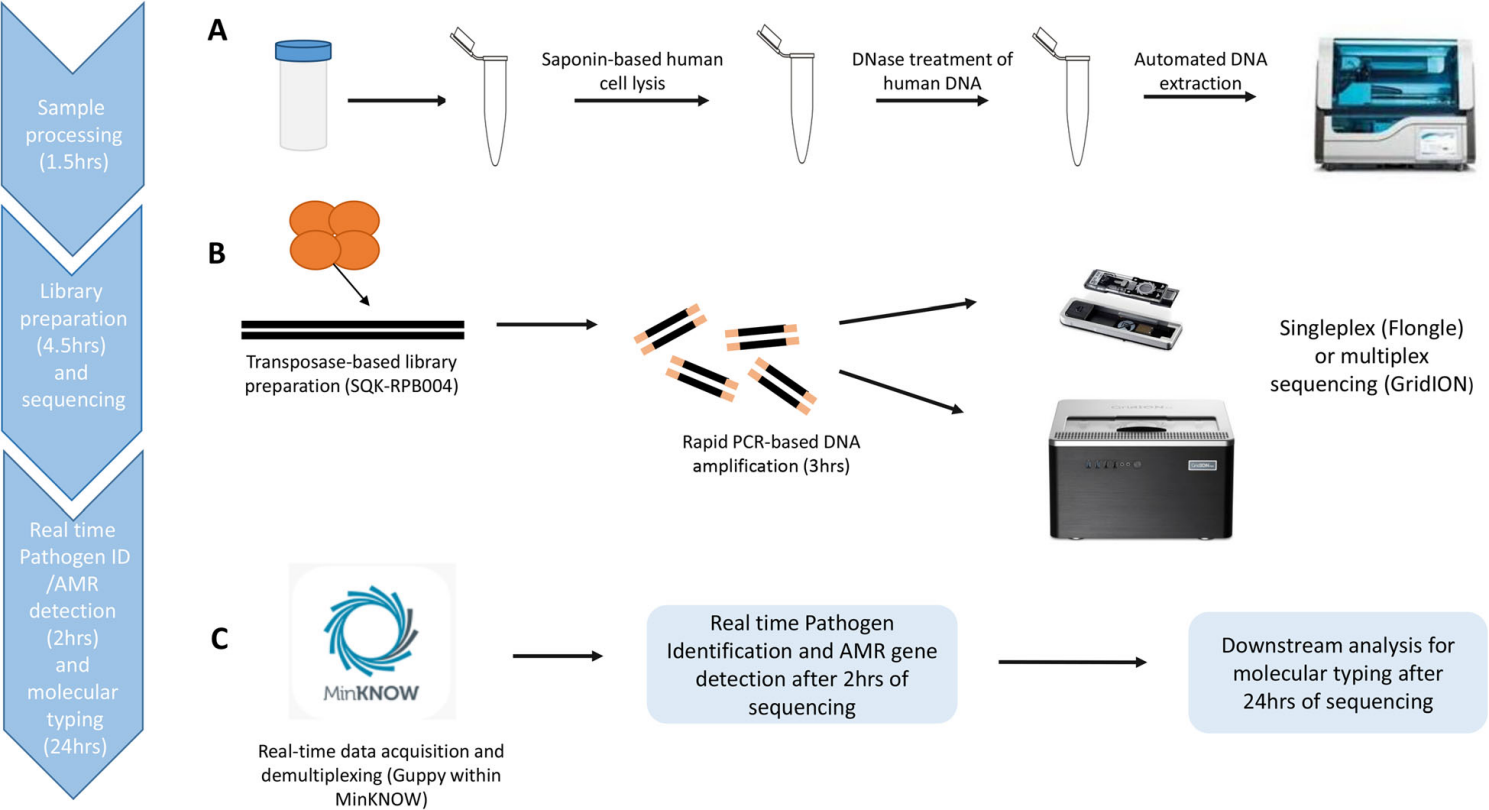
Schematic workflow representing the main steps of the CMg workflow. **A.** Sample processing of respiratory samples during which sample is treated with saponin to lyse human cells followed by nuclease treatment of human DNA and microbial cells are bead-beaten and automated microbial NA extraction is carried out. **B.** DNA is prepared for nanopore sequencing using the Rapid PCR Barcoding kit (SQK-RPB004), then the library is sequenced either with a Flonge (1 sample only) or with a GridION (6 samples). **C.** Real-time data acquisition is carried out by MinKNOW during which squiggle plots are converted into raw sequencing data and low-quality reads are removed (qscore = > 6). Real-time basecalling and demultiplexing (multiplex runs only) of raw data are done simultaneously by Guppy. Human reads (if any) are then firstly subtracted via read-based alignment offline; pathogen identification (ID) and AMR gene detection are then followed in real-time after 2 h of sequencing. *K*-mer-based classification is used for microbial ID (WIMP within EPI2ME), and offline read-based alignment for AMR gene detection based on pathogen/s (if any) identified by the previous step (above pre-defined thresholds) is followed by Scagaire. After 24 h of sequencing, downstream offline analysis can be carried out (if needed) for molecular typing to characterised identified pathogens for public health purposes

Clinical metagenomics (CMg) using nanopore sequencing has the potential to meet these needs due to its unbiased pan-microbial coverage and ability to provide real-time analysis [9]. CMg has been evaluated for respiratory, urinary tract and prosthetic joint infections [9–12]; however, its ability to simultaneously provide rapid results informing antimicrobial treatment and infection control decisions has only been demonstrated in a few studies [13]. We therefore performed a proof-of-concept study using a saponin-based, previously published CMg pipeline [9] with a slight modification—depletion reaction was performed at 37 °C instead of room temperature (workflow outlined in Fig. 1). CMg was used on respiratory specimens from COVID-19 patients with suspected secondary bacterial or fungal pneumonia. The objective was to assess whether rapid CMg testing had the potential to inform initial antimicrobial treatment decisions and rapidly detect outbreaks in an expanded COVID-19 intensive care setting.

## Methods

### Clinical setting and data collection

Clinical, microbiological and ward location data were collected by the primary care team from all intubated patients with a documented SARS-CoV-2 RT-PCR positive test admitted to the 3 pre-existing ICUs with expanded capacity and 4 newly opened COVID-19 ICUs at St Thomas’ Hospital, London. All healthcare staff used additional personal protective equipment (PPE) according to Public Health England (PHE) guidelines. Updated ICU empiric antimicrobial guidelines recommended 3 days co-amoxiclav for COVID-19 patients on admission, piperacillin-tazobactam for first suspected ICU-acquired respiratory infection and meropenem for subsequent infections or where resistance was suspected.

### Sample selection and analysis

Between 11 April and 15 June 2020, surplus clinical respiratory samples from 34 ICU COVID-19 patients with suspected secondary infections were processed by the research team after routine processing. Samples processed by the clinical laboratory included respiratory clinical samples (tracheal aspirates, bronchoalveolar lavages (BALs) and non-direct bronchoalveolar lavages (NDLs, a BAL collected without the use of a bronchoscope) for (i) routine microbiological culture for bacterial and fungal pathogens or detection of SARS-CoV-2 by PCR and (ii) sera and BALs for galactomannan (GM) antigen detection when *Aspergillus* infection was suspected (described further below). Surplus of samples subjected to routine microbiology culture for bacterial and fungal pathogens was only collected by the research team for CMg processing after routine testing was performed. In total, 43 surplus samples were collected which included 10 BALs, 6 tracheal aspirates and 27 NDLs. These were used to assess the performance of the CMg workflow which included rapid bacterial and fungal identification, AMR gene detection and pathogen genomic epidemiology (Fig. 1). Samples were anonymised prior to submission to the research team. The clinical care team collected relevant clinical and laboratory data to create an anonymised dataset given to the research team who had no access to patient identifiable data at any time. The intensive care clinical team was not aware of the CMg results whilst caring for the patients. Collected samples were stored (1–4 days) at 4 °C until processed aseptically. The full process for sample collection, nanopore sequencing, data linkage and anonymization was approved by a research ethical committee (North West Preston REC: reference 18/NW/0584).

### Routine microbiological processes

Routine processing of respiratory samples was initially performed in an ISO15189-accredited laboratory according to standard operating procedures [14]. Briefly, BALs and NDLs were centrifuged at 1200*g* for 10 min, and the supernatant was discarded leaving 500 μl residual volume. The remaining sample was resuspended (vortex for 10 s), and 10 μl of sample was streaked onto blood agar, chocolate agar and fastidious anaerobic agar (FAA). Tracheal aspirates were not centrifuged and were directly streaked onto blood agar and chocolate agar plates. All plates were then incubated at 37 °C in an aerobic and an anaerobic environment for 48 h. Sabouraud agar plates were set up for the detection of *Candida* spp. and *Aspergillus* spp. and incubated for 5 days at 37 °C in aerobic conditions. Bacterial colonies were identified using MALDI-TOF (Bruker) except the *Aspergillus* spp. where microscopy was performed. Culture-negative samples were reported as ‘normal respiratory flora (NRF)’ or as ‘no growth (NG)’ when no organisms would be observed after 48 h of incubation.

Antibiotic susceptibility by agar diffusion was performed for any reported grown pathogens, following guidelines of the European Committee on Antimicrobial Susceptibility Testing (EUCAST) methodology [15].

### Reporting of respiratory pathogens from CMg data

Microorganisms referred to as ‘respiratory pathogens’ or ‘pathogens’ in this study were defined as common agents causing respiratory infection in ICU patients. A pre-defined pathogen list was compiled based on previous lower respiratory tract infections studies [9, 16–18] (listed in Additional file 1: Table S1). Respiratory pathogens identified in samples tested in this study were *Acinetobacter baumanni*, *Aspergillus fumigatus*, *Bulkhoderia* spp., *Citrobacter koseri*, *Citrobacter freundii*, *Enterobacter cloacae complex*, *Klebsiella aerogenes*, *Klebsiella oxytoca*, *Klebsiella pneumoniae*, *Morganella morganii*, *Proteus mirabilis*, *Pseudomonas aeruginosa*, *Serratia marcescens*, *Stenotrophomonas maltophilia* and *Staphylococcus aureus*. *Haemophilus influenzae* was identified in one negative process control only. *Corynebacterium striatum* was not considered a pathogen but was only investigated in our study for molecular typing due to the increased number of incidence of the organism in ICU during the study. Microorganisms identified in this study (above chosen thresholds) but not defined as respiratory pathogens are listed in Additional file 1: Table S2.

### Routine SARS-CoV-2 RT-PCR

For routine detection of SARS-CoV-2, reverse-transcriptase (RT) PCR was performed by the clinical laboratory using the Highplex 24 system (AusDiaganostics Pty Ltd.), according to the manufacturer’s instructions (SARS-CoV-2, Influenza and RSV 8-well, Catalogue number: 20081, version: 08), which targets the Orf1ab and Orf8 of SARS-CoV-2 and requires 200 μl of respiratory clinical samples.

### Galactomannan assay

For GM antigen detection, the clinical laboratory sent referred samples to the Mycology Reference Laboratory National Infection Services, PHE at Southmead Hospital, Bristol. The Platelia Aspergillus Antigen kit (BIO-RAD – 62794) was used according to the manufacturer’s instructions to detect GM in the sera and BALs only. The assay is a one-stage immunoenzymatic sandwich microplate and uses rat EBA-2 monoclonal antibodies designed to detect *Aspergillus* GM antigens in clinical samples.

### *A. fumigatus* qPCR assay

A probe-based qPCR assay was performed on all samples from the CMg cohort (*n* = 43) to detect *A. fumigatus* DNA (previously described in [19]). The assay was done using the QuantStudio 7 Flex (Applied Biosystems). The master mix for each reaction consisted of 10 μl of LightCycler 480 probe master (2×), 0.4 μl of probe (final concentration 0.2 μM) and 0.5 μl each of the forward and reverse primer (final concentration 0.25 μM); 2 μl of DNA was added, and nuclease-free water was added to the reaction to make the volume up to 20 μl. The qPCR conditions were pre-incubation at 95 °C for 15 min and amplification for 40 cycles at 94 °C for 15 s and 60 °C for 1 min.

### Nanopore metagenomic sequencing

Host DNA depletion, microbial DNA extraction and sequencing were performed based on previously published methods [9]. Briefly, collected surplus respiratory samples were sputasol-treated (SR0233 - Oxoid) in a 1:1 ratio for 15 min at 37 °C to liquefy samples before treatment with working stock of 1% saponin (15 min at 37 °C shaking at 1000 rpm; Sigma – 47036-50G-F) to induce host cell lysis and release of host DNA that was digested with HL-SAN DNase (15 min at 37 °C shaking at 1000 rpm; Articzymes – 70910-202). Samples were then washed twice in 1.5 μl PBS and centrifuged to pellet bacterial and fungal organisms. The pellet was re-suspended in lysis buffer (600 μl; Qiagen UK) for bead-beating (Lysis Matrix E beads and 1 min at 50 o/s on FastPrep 24; MP Biomedical) to release microbial DNA followed by centrifugation (1 min at top speed in benchtop centrifuge) and removal of ~ 200 μl supernatant. The supernatant was then proteinase K-treated (5 min at 65 °C shaking at 1000 rpm; Qiagen) to digest residual proteins. Finally, samples were incubated at 95 °C for 30 min to kill residual organisms before DNA extraction using the Fast Pathogen 200 protocol on a MagNA Pure 24 System (Roche UK). DNA was quantified using the high sensitivity dsDNA assay kit (Thermo Fisher) on the Qubit 3.0 Fluorometer (Thermo Fisher). Fragment size and quality of metagenomic libraries were analysed using the TapeStation 4200 (Agilent Technologies) automated electrophoresis platform.

Samples were batched for CMg sequencing (6 samples per run) plus a negative process control. In the negative process control, sample was replaced with water and processed through the full pipeline including human DNA depletion, DNA extraction library preparation, sequencing and analysis. This control was introduced to monitor barcode cross-talk and laboratory and/or reagent contamination. Library preparation was performed using the Rapid PCR Barcoding Kit (ONT) as previously described [9] but with a 6-min PCR extension time. Library was loaded onto nanopore flow cells (R9.4.1) with sequencing performed on the GridION platform. The ONT MinKNOW software (version 3.6.5) acquired raw sequence data with live basecalling by ONT Guppy (version 3.2.10). Sequencing was run for 24 h with the first 2 h data used for pathogen identification by WIMP analysis. Human reads were discarded by alignment with genome reference (GCA_000001405.15, assembly GRCh38.p13 version) and non-human reads were exported and used for pathogen identification and AMR gene detection as previously described [20] (see Fig. 1 for a schematic workflow of the CMg method).

### Pathogen identification and resistance gene prediction

The EPI2ME Antimicrobial Resistance pipeline (ONT, version v2020.2.10-3247478) was used for bacterial and fungal pathogen identification as previously described [9]. The EPI2ME Antimicrobial Resistance pipeline uses What’s In My Pot (WIMP), for the identification of respiratory bacterial and fungal pathogens. WIMP (v3.4.0) uses ‘Centrifuge’ a *k-*mer-based metagenomic classifier [20] and a pre-built database containing 56,044 sequences, which is based on the NCBI taxonomy and RefSeq database [21] but is further curated by ONT to remove low-complexity sequence regions (protocol available at https://figshare.com/articles/online_resource/Additional_file_3/16722829/1) [22].

Potential bacterial pathogen(s) were reported if they represented ≥ 1% of total microbial reads with a centrifuge score ≥ 2504 as a quality threshold. *Aspergillus* spp. were only reported if ≥ 10 reads (with a centrifuge score ≥ 2504) were identified. To remove barcode cross-talk between samples on the multiplexed runs, 0.1% of pathogen reads were removed from all samples (i.e. from each barcode) if there were > 10,000 cumulative pathogen reads identified from the 6 samples on the flow cell. Any pathogens identified in the negative control (with > 5 classified reads) after application of all thresholds were considered contaminants, and these pathogens were removed from all multiplexed samples on the sequencing run.

Thresholds used in this study for pathogen identification were defined using the dataset published by Charalampous et al. [9] as the training set (see Additional file 1: Supplementary methods).

Sensitivity and specificity were calculated on a per-sample basis [14] using the Clopper–Pearson exact method (https://www.medcalc.org/calc/diagnostic_test.php).

Resistance genes were detected from 2 h of sequencing using Scagaire with default parameters. Scagaire utilises a bundled database containing the 40 most common-sequenced bacterial species in the RefSeq database and only reports clinically relevant resistance genes [23]. Briefly, FASTQ files were converted into FASTA files and then analysed using Abricate [24], with default parameters, to detect resistance genes against the ResFinder database. Then, Scagaire was used to predict and filter out clinically relevant genes based on the pathogen identified by metagenomics and the Abricate output file. Clinically relevant gene alignments with < 90% coverage were removed and only resistance genes with > 1 gene alignment were reported to remove any possible bioinformatics errors.

This analysis was only carried out to determine the presence or absence of genotypic determinants conferring resistance to antibiotics used on the ICU for GNB and *Staphylococcus aureus*. Furthermore, analysis was only performed where there was concordance between organisms identified in both routine culture and CMg, so that genotypic-determinants and culture results could be directly compared. Samples where *Pseudomonas aeruginosa* was identified as the sole pathogen were excluded, due to known difficulty in predicting phenotypic resistance based on genotypic elements only [25, 26].

### DNA extraction and nanopore sequencing of *K. pneumoniae* BSI isolates

Isolates of *K. pneumoniae*, previously identified by MALDI-TOF, were subcultured on blood agar and incubated for 48 h at 35 °C aerobically. For bacterial DNA extraction, 4–5 colonies were selected and were mixed in 500 μl of PBS. The mixed solution was transferred into Lysing Beads - Matrix E (MP Biomedicals - 116005500) and bead-beaten for 4 m/s for 40 s seconds using a MP Biomedicals FastPrep-24 5G Instrument (MP Biomedicals - 116005500). The sample was then centrifuged for 1 min at 12,000 rpm, and 100 μl of the supernatant was collected and transferred to a clean 1.5-ml Eppendorf tube. Then, extracted DNA was then subjected to a bead wash to remove short DNA fragments. Briefly, 0.5× of Agencourt AMPure XP beads (Beckman Coulter-A63881) was added, mixed and incubated for 10 min at RT. The tube was placed in a magnetic rack and washed twice with 80% of ethanol before the sample was eluted in 50 μl of nuclease-free water.

Next, library preparation for nanopore sequencing was done, using the native barcoding genomic DNA (ONT - EXP-NBD114 and SQK-LSK109 kits). Isolates were sequenced on a GridION for 48 h, following the manufacturer’s instructions.

### *Klebsiella* spp. and *C. striatum* SNP analysis

Representative complete reference genomes for each species were downloaded from RefSeq to generate consensus sequences [27]. *K. pneumoniae* reads from 7 patients (8 samples) were aligned to the *K. pneumoniae* subsp. pneumoniae HS11286 strain. *K. aerogenes* reads from 4 samples (3 patients) were aligned to the *K. aerogenes* strain NCTC9735. *C. striatum* reads in 5 samples (4 patients) were aligned to *C. striatum* strain KC-Na-01. Reads were aligned to each matching reference genome using minimap2 (v 2.17-r941) [28]. A consensus sequence was generated using bcftools (v 1.10.2) [29]. SNP-sites (v2.5.1) [30] was used to identify SNPs between each sample, and SNP distances were calculated using SNP-dists (v0.7.0) (https://github.com/tseemann/snp-dists). Multi-locus sequence typing was performed using mlst (v2.19.0) [31]. FASTQ/FASTA files were transformed using PyFASTAQ (v3.17.0) (https://github.com/sanger-pathogens/Fastaq). SNP distances were calculated using SNP-dists (v0.7.0) (https://github.com/tseemann/snp-dists). Genomes with a genetic similarity of ≥ 99.99% were considered related, and plausible outbreaks were investigated using traditional epidemiological methods. The threshold for genetic similarity was based on previous studies [32, 33] and the latest nanopore accuracy data (https://nanoporetech.com/accuracy).

## Results

### Clinical and microbiological characteristics of COVID-19 patients

In total, 175 invasively ventilated COVID-19 patients were admitted to 7 ICUs, between 11 April and 15 June 2020, of which 34 patients with suspected secondary infection were chosen for inclusion in this CMg proof-of-concept study and had one or more respiratory samples analysed by CMg (Table 1). Admission characteristics of the CMg group were broadly comparable to those not receiving CMg testing, with a median age of 52 and 70% being male, although they had a longer median length of hospital-stay (32 days [IQR 24–47] compared to 25 days [IQR 15–45] in the non-CMg group). The 34 CMg patients had 156 respiratory samples collected with organisms identified by routine cultures from at least one sample. The main respiratory sample Gram-negative bacteria were *Klebsiella* spp. (53%), *Citrobacter* spp. (15%) and *E. coli* (9%). The main Gram-positive bacteria were *S. aureus* (9%), *C. striatum* (24%) and *Enterococcus* spp. (12%). *C. albicans*, other *Candida* spp. and *Aspergillus* spp. were cultured from 38%, 15% and 9% of patients, respectively. Respiratory pathogens cultured from CMg patients were representative of those found in the samples from the patient cohort over the 9-week period of the CMg study (Table 1) as well as with patients admitted across the 7 ICUs during the first wave from March to June 2020 (Additional file 1: Table S3).

**Table 1:** Clinical characteristics and results of routine microbiological tests performed on intubated COVID-19 patients during the CMg study across 7 linked dedicated COVID-19 intensive care units on Guy’s and St Thomas’ Hospital sites

	Non-metagenomics group (***n*** = 141)	Metagenomics group^**a**^ (***n*** = 34)
Median age (IQR)	56 (46–61)	52 (41–58)
Sex–male	101 (72%)	23 (70%)
Ethnicity		
White	49 (35%)	16 (47%)
Black and minority ethnicities	74 (52%)	15 (44%)
Not known	19 (13%)	3 (9%)
Mortality	34 (25%)	8 (24%)
Length of stay (IQR)	25 days (15–45)	32 days (24–47)
**Respiratory cultures in ICU**^b^		
Median samples per patient (IQR)	2 (1–3)	4 (4–6)
Total number of samples/patients tested	372/117	180/34
**Organisms from respiratory culture whilst in ICU (number of individuals who ever had the following organisms in any sample)**		
*Klebsiella* spp.	48 (34%)	18 (53%)
*Staphylococcus aureus*	14 (10%)	3 (9%)
*Citrobacter* spp.	14 (10%)	5 (15%)
*Escherichia coli*	7 (5%)	3 (9%)
*Pseudomonas* spp.	10 (7%)	1 (3%)
*Corynebacterium striatum*	8 (6%)	8 (24%)
*Enterococcus* spp.	12 (9%)	4 (12%)
*Serratia* spp.	9 (6%)	2 (6%)
*Enterobacter* spp.	6 (4%)	1 (3%)
*Haemophilus* spp.	2 (1%)	0 (0%)
*Stenotrophomonas maltophilia*	4 (3%)	1 (3%)
*Proteus* spp.	0 (0%)	4 (12%)
*Morganella* spp.	0 (0%)	1 (3%)
*Acinetobacter* spp.	0 (0%)	1 (3%)
*Streptococcus pyogenes*	3 (2%)	0 (0%)
*Candida albicans*	40 (28%)	13 (38%)
*Candida* spp. (non-*albicans*)	10 (7%)	5 (15%)
*Aspergillus* spp.	1 (1%)	3 (9%)
No organisms isolated	40 (30%)	2 (6%)
**Galactomannans (GMs)**		
**Bronchoalveolar lavage (BAL) GMs**		
Number of tests/patients tested	38/28	25/16
Positive tests/patients positive	0/0	6/5
**Serum GMs**		
Number of tests/patients tested	74/50	34/22
Positive tests/patients positive	4/4	3/3

^a^One patient was SARS-CoV-2 RNA PCR-negative but had clinical diagnosis of COVID-19

^b^One patient in the metagenomics group and 48 patients from the non-metagenomics group had no respiratory specimens collected whilst on ICU during the study period

### Performance of CMg compared with routine culture for pathogen detection

Potential respiratory pathogens were cultured from 26/43 (60%) samples (18 NDLs, 4 BALs and 4 tracheal aspirates) tested by CMg (Fig. 1) with 17 samples (9 NDLs, 6 BALs and 2 tracheal aspirates) reported by culture either as no growth or not containing any pathogenic organisms (Table 2). CMg identified 24/26 culture-reported pathogens (92% sensitive; 95% CI, 75–99%) using pre-defined criteria (i.e. ≥ 1% of microbial classified reads with a centrifuge score ≥ 2504) (Table 2). Metagenomics did not report *K. aerogenes* in two polymicrobial samples (S44 and S45) where scanty growth of *K. aerogenes* was reported by culture (S45 was also culture-positive for *C. striatum*). *K. aerogenes* reads were identified in both samples by CMg sequencing but were below pre-defined thresholds (Additional file 1: Table S4A). Applying the barcode cross-talk threshold did not affect pathogen identification in samples but allowed identification of contaminants in negative controls—*E. coli* was the most common contaminant identified in 8/11 negative controls (Additional file 1: Table S4B).

**Table 2:** Comparison of pathogens reported by routine culture with metagenomics sequencing in respiratory samples

Patient ID	Sample ID	Semi-quantitative routine culture report^a^	Pathogens identified by metagenomic sequencing	Pathogen reads^b^ identified by metagenomic sequencing	Microbial reads^b^ identified by metagenomic sequencing
26	S35	*Acinetobacter* spp. (L)	*A. baumannii*	99	853
100	S39	*C.koseri* (H)	*C. koseri* *K. pneumoniae*	23,870 284	26,251
121	S37	*P.mirabilis* (M) *M.morganii* (M)	*P. mirabilis* *M morgannii* *K. pneumoniae*	397 28,300 876	45,395
177	S36	*S. aureus* (H)	*S. aureus*	109,767	119,881
196	S42	*B. cenocepacia* (L)	*Burkholderia* spp.	34,347	51,551
400	S49	*K. pneumoniae* (M)	*K. pneumoniae*	594	2202
408	S20	*S. aureus* (M)	*S. aureus*	36,281	38,708
441	S21	*E. cloacae* (M)	*E. cloacae*	62,314	75,866
	S51	*S. aureus* (L) *C. koseri* (L)	*S. aureus* *C. koseri*	5203 2262	8582
550	S10	*K. pneumoniae* (M)	*K. pneumoniae*	69,029	75,350
563	S28	*Aspergillus* (S)	*A. fumigatus* *S. aureus*	2649 3165	44,684
613	S18	Negative	Negative	0	22,776
618	S45	*K. aerogenes* (S)	–	104	1228
677	S52	*K. aerogenes* (L)	*K. aerogenes*	5277	38,854
	S54	Negative	Negative	1758	3015
	S63	*K. pneumoniae* (M)	*K. pneumoniae*	17,034	147,379
727	S53	Negative	Negative	0	0
740	S30	Negative	Negative	0	759
	S59	*K. pneumoniae* (M)	*K. pneumoniae*	99,186	119,458
749	S40	Negative	Negative	0	1157
	S62	*K. aerogenes* (L)	*K. aerogenes*	184	1021
815	S25	Negative	Negative	0	1413
	S46	*C. koseri* (M)	*C. koseri*	237	462
855	S41	Negative	*S. aureus*	1365	31,067
872	S11	*K. pneumoniae* (H)	*K. pneumoniae*	16,828	35,668
	S61	*P. mirabilis* (H) *K. pneumoniae* (M)	*P. mirabilis* *K. pneumoniae* *C. koseri*	29,797 14,118 815	47,924
1033	S8	*A. fumigatus* (S)	*A. fumigatus* *K. oxytoca*	77 44	1399
1036	S5	Negative	Negative	0	0
1054	S31	*K. pneumoniae* (L)	*K. pneumoniae*	28,056	31,800
1065	S16	Negative	*S. aureus*	1768	43,692
	S19	Negative	Negative	0	10,371
1069	S17	*P. aeruginosa* (M)	*P. aeruginosa*	1457	1905
1082	S14	Negative	Negative	0	205
1092	S27	Negative	Negative	0	1413
1262	S29	Negative	Negative	0	759
1292	S44	*S. marcescens* (L) *K. aerogenes* (S)	*S. marcescens* *C. freundii*	53,082 6082	65,078
1346	S56	*A. fumigatus* (S) *P. mirabilis* (L)	*A. fumigatus* *P. mirabilis*	79 11,323	36,658
1440	S33	Negative	Negative	0	1176
1457	S64	Negative	Negative	0	24,484
	S65	Negative	Negative	0	45,990
1503	S1	*K. aerogenes* (L)	*K. aerogenes*	138,626	145,195
1512	S34	*K. pneumoniae* (L)	*K. pneumoniae*	38,758	82,796
1538	S55	Negative	*A. fumigatus*	16	9146

^a^Reported growth by culture for each pathogen. *H=* heavy growth, *M* = moderate growth, *L =* light growth, *S* scanty growth

^b^Criteria for reporting organisms was ≥ 1% of microbial-classified reads and with a centrifuge score ≥ 2504 and > 9 reads for *A. fumigatus* only

CMg identified 6 additional pathogens in 6 culture-positive samples (3 *Klebsiella* spp., 1 *S. aureus*, 1 *C. koseri* and 1. *C. freundii*) (Table 2). Three of these organisms were identified by culture in other respiratory samples from those patients (*K. oxytoca* (S8), *K. pneumoniae* (S37) and *C. koseri* (S61)). CMg also reported 3 additional pathogens in 3/17 culture-negative samples (one *A. fumigatus* (S55) and two *S. aureus* (S16 and S41)). The *A. fumigatus* was from a patient that had positive serum GM and culture-positive *A. fumigatus* respiratory samples that were not tested in this study (Table 4) resulting in a specificity of 82% (95% CI, 57–96%) based on culture-negative samples only. Considering additional species identified by CMg only (*n* = 9/43 samples) as ‘false-positive findings’, specificity was 79% (95% CI, 64–90%). Note that specificity and sensitivity were calculated on a per-sample basis; hence, culture-positive samples with additional pathogens reported by CMg only were not considered as false-positives and only culture-positive samples where all culture-reported pathogens in the sample were also detected by CMg were considered true-positives (sample numbers were too small to analyse on a per-pathogen basis).

### Impact of resistance gene detection on guideline-directed empiric beta-lactam antibiotic selection

Two-hour CMg sequencing data was analysed from 20 of 26 culture-positive samples where the presence of resistance genes could predict phenotypic resistance and impact on guideline-directed beta-lactam treatment (Table 3). This analysis included (i) samples positive for *Enterobacterales* or *Acinetobacter* spp. where the presence of beta-lactam resistance could change advice on first-line beta-lactam treatment and (ii) presence of *mecA* genes in *S. aureus* culture-positive samples. The remaining 6 culture-positive samples were not positive for *Enterobacterales* and were not included in this analysis.

**Tab﻿le 3:** Comparison of CMg-identified genotypic resistance with phenotypic culture results and the impact on guideline-recommended beta-lactam antibiotic treatment

Sample ID	Bacteria reported by culture and metagenomics	Culture-reported resistance	CMg predicted resistance	Relevant genes identified	Genotype/phenotype match?	CMg-based treatment recommendation^a^
S1	*K. aerogenes*	No	–	–	Y	Meropenem
S10	*K. pneumoniae*	No	–	–	Y	Co-amoxiclav
S11	*K. pneumoniae*	No	–	–	Y	Co-amoxiclav
S20	*S. aureus*	Erythromycin	Erythromycin	*erm*	Y	Co-amoxiclav
S21	*E. cloacae*	No	No	–	Y	Meropenem
S31	*K. pneumoniae*	ESBL Co-trimoxazole	ESBL Co-trimoxazole	*bla*_TEM_, *bla*_SHV_ *bla*_CTX-M_, *sul*	Y Y	Meropenem
S34	*K. pneumoniae*	No	ESBL	*bla*_TEM,_ *bla*_SHV_	N	Meropenem^b^
S35	*A. baumannii*	ESBL	–		N	Meropenem
S36	*S. aureus*	Erythromycin Trimethoprim	Erythromycin Trimethoprim	*erm* *dfrG*	Y	Co-amoxiclav
S37	*P. mirabilis*	No	Amoxicillin Trimethoprim	*bla*_OXA_ *dfrA*	N N	Meropenem
	*M. morganni*	Co-trimoxazole Fosfomycin Nitrofurantoin	Co-trimoxazole – –	*dfrA*	Y N N	
S39	*C. koseri*	Amoxicillin	Amoxicillin	*bla*_CKO_	Y	Co-amoxiclav
S44	*S. marcescens*	No	–	–	Y	Meropenem
S49	*K. pneumoniae*	ESBL	ESBL	*bla*_TEM_	Y	Meropenem
S51	*S. aureus*	Erythromycin	Erythromycin	*erm*	Y	Co-amoxiclav
	*C. koseri*	Amoxicillin	Amoxicillin	*bla*_CKO_	Y	
S52	*K. aerogenes*	Gentamicin	–	–	N	Meropenem
S56	*P. mirabilis*	Amoxicillin Co-trimoxazole	Amoxicillin –	*bla*_TEM_ –	Y N	Co-amoxiclav
S59	*K. pneumoniae*	ESBL Co-trimoxazole	ESBL Co-trimoxazole	*bla*_TEM_, * bla*_SHV_, *sul*	Y Y	Meropenem
S61	*P. mirabilis*	No	–	–	Y	Co-amoxiclav
	*K. pneumoniae*	No	–	–	Y	
S62	*K. aerogenes*	ESBL	–	–	N	Meropenem
S63	*K. pneumoniae*	ESBL	ESBL	*bla*_SHV_	Y	Meropenem

^a^Recommended antibiotics are those defined in the Guy’s and St Thomas’ Guideline for empiric and targeted first-line treatment for ITU-acquired ventilator-associated pneumonia (VAP). Piperacillin-tazobactam is the first line empiric choice with recommendation to change therapy based on culture results and discussion with microbiology and infectious diseases. Meropenem is used for ESBL-Enterobacterales and *E. cloacae*, *K. aerogenes* (formally *E. aerogenes*), *M. morganii* and *S. marcescens* that have inducible β-lactam resistance. Co-amoxiclav is recommended for susceptible organisms

^b^Detection of ESBL by metagenomics for *K. pneumoniae* in this sample was not confirmed by culture

There was concordance between genotypic CMg, and the reported phenotypic beta-lactam resistance in all but one sample. Extended-spectrum β-lactamase (ESBL) genes were detected in 4 samples containing *K. pneumoniae* that was phenotypically reported as an ESBL (Table 3). These included ESBL *bla*_TEM_ genes identified in samples S49, S59 and S31. Additionally, *bla*_SHV_ and *bla*_CTX-M_ genes were identified in S31 and S59. In S63, culture reported a co-amoxiclav- and piperacillin-tazobactam-resistant *K. pneumoniae*, and a *bla*_SHV_ gene was identified by CMg possibly explaining the reported phenotype.

No β-lactam resistance genes were found in 8 samples containing 9 susceptible *Enterobacterales* (Additional file 1: Table S5), but *bla*_TEM_ and *bla*_SHV_ genes were detected in a sample with *K. pneumoniae* having no reported phenotypic resistance (S34). Resistance phenotypes could not be genotypically predicted in two samples with light bacterial growth of *A. baumanni* (S35) and *K. aerogenes* (S62) due to low read count by metagenomic sequencing. No carbapenemases were detected in any sample, and no SCC_mec_ elements were found in the two samples growing *S. aureus*, consistent with the reported phenotypes.

Identified genes conferring resistance against non-guideline recommended antibiotics were all consistent with reported phenotypes (Table 3). These included erythromycin resistance in two *S. aureus* samples (S20 and S51) where *erm* genes were reported, plus erythromycin and trimethoprim resistance in one *S. aureus* sample (S36) where *erm* and *dfrG* genes were detected. Additionally, *sul* genes were detected in three co-trimoxazole resistant GNB-positive samples (S31, S37 and S59), possibly explaining the reported phenotype (Table 3).

The potential impact of CMg data was assessed against the guideline-recommended first-line empirical antibiotic treatment for VAP (piperacillin-tazobactam). CMg results would recommend meropenem rather than piperacillin-tazobactam in 11/20 cases, based on speciation in 7 (35%) and resistance-gene detection in 4 (20%), and co-amoxiclav in 8 cases rather than piperacillin-tazobactam, based on speciation combined with the absence of β-lactamase genes (40%). In 1/20 (5%) cases, CMg directed antibiotic choice was not consistent with culture (S34) where identification of an ESBL was not phenotypically reported by culture.

### Comparison of methods for diagnosis of IPA

GM antigen detection tests were requested on BAL and serum samples from 16 (47%) and 22 (65%) patients, respectively, from the CMg group (Table 1). Nine patients had at least one mycology result consistent with IPA (Table 4). Four of five culture-positive patients met the original AspICU criteria, and all met the modified AspICU criteria that do not require predisposing host factors [34, 35]. Two-hour CMg sequence data identified *A. fumigatus* reads in all of the 3 culture-positive samples that were tested by CMg (S8 [77 reads], S28 [2649 reads] and S56 [79 reads]). Four persistently culture-negative patients had positive BAL-GM and met the modified AspICU criteria [4]; none of these had *Aspergillus* detected by CMg or qPCR (Table 4).

**Table 4:** Mycological tests and clinical characteristics of patients with at least one result suggestive of invasive pulmonary Aspergillosis

Patient	***A. fumigatus*** detection in respiratory samples				Galactomannan (positive/tested)		AspICU – Putative Criteria [34]			ECMO
	Sample number^a^	CMg	qPCR (Cq)	Culture (Positive/Tested)	BAL > 1.0	Serum > 0.5	Radiology	Clinical	Host	
563	S28	Positive	31	Positive			Yes	Yes	Yes – steroid	No
	Other	ND	ND	3/6	0/1	0/0				
613	S18	Negative	> 40	Negative			Yes	Yes	No	No
	Other	ND	ND	2/2	1/1	0/0				
677	S63	Negative	> 40	Negative			Yes	Yes	Yes – steroid	No
	S54	Negative	> 40	Negative						
	S52	Negative	> 40	Negative						
	Other	ND	ND	0/8	2/2	0/5				
740	S59	Negative	> 40	Negative			Yes	Yes	Yes – leukaemia on chemotherapy	No
	S30	Negative	> 40	Negative						
	Other	ND	ND	0/16	1/4	1/2				
1033	S8	Positive	33	Positive			Yes	Yes	Yes – steroid	Yes
	Other	ND	ND	0/0	1/1	1/1				
1346	S56	Positive	32	Positive			Yes	Yes	Yes – steroid, anakinra	Yes
	Other	ND	ND	0/3	1/1	0/2				
1440	S33	Negative	> 40	Negative			Yes	Yes	Yes – steroid, anakinra	Yes
	Other	ND	ND	0/4	1/2	0/1				
1457	S65	Negative	> 40	Negative			Yes	Yes	Yes – steroid	Yes
	S64	Negative	> 40	Negative						
	Other	ND	ND	0/9	2/2	0/2				
1538	S55	Positive	31	Negative			Yes	Yes	Yes – lymphoma on chemotherapy	No
	Other	ND	ND	4/5	0/0	1/1				

ND = not done

^a^Other sample represents samples from the nine patients, retrieved after an ITU episode but were outside the CMg period and were not processed with CMg

CMg detected *A. fumigatus* in a sample from a patient with *A. fumigatus* in other diagnostic samples (S55 [16 reads]) (Table 2). Probe-based qPCR was 100% concordant with CMg (Table 4). One sample from a patient (S18) growing *A. fumigatus* in additional tested samples was negative by culture, qPCR and metagenomic sequencing. CMg did not report any *Aspergillus* reads in the remaining culture-negative samples and was concordant with qPCR and culture (Additional file 1: Table S6).

Post-mortem histology from patient 563 with *A. fumigatus* identified by culture and CMg revealed a single 1 cm × 1 cm patch of IPA and no *A. fumigatus* in other organs. There was extensive diffuse alveolar damage, and IPA was not reported to have contributed to death (Additional file 2: Figure S1).

### CMg detection of hospital transmission

**Fig. 2:**
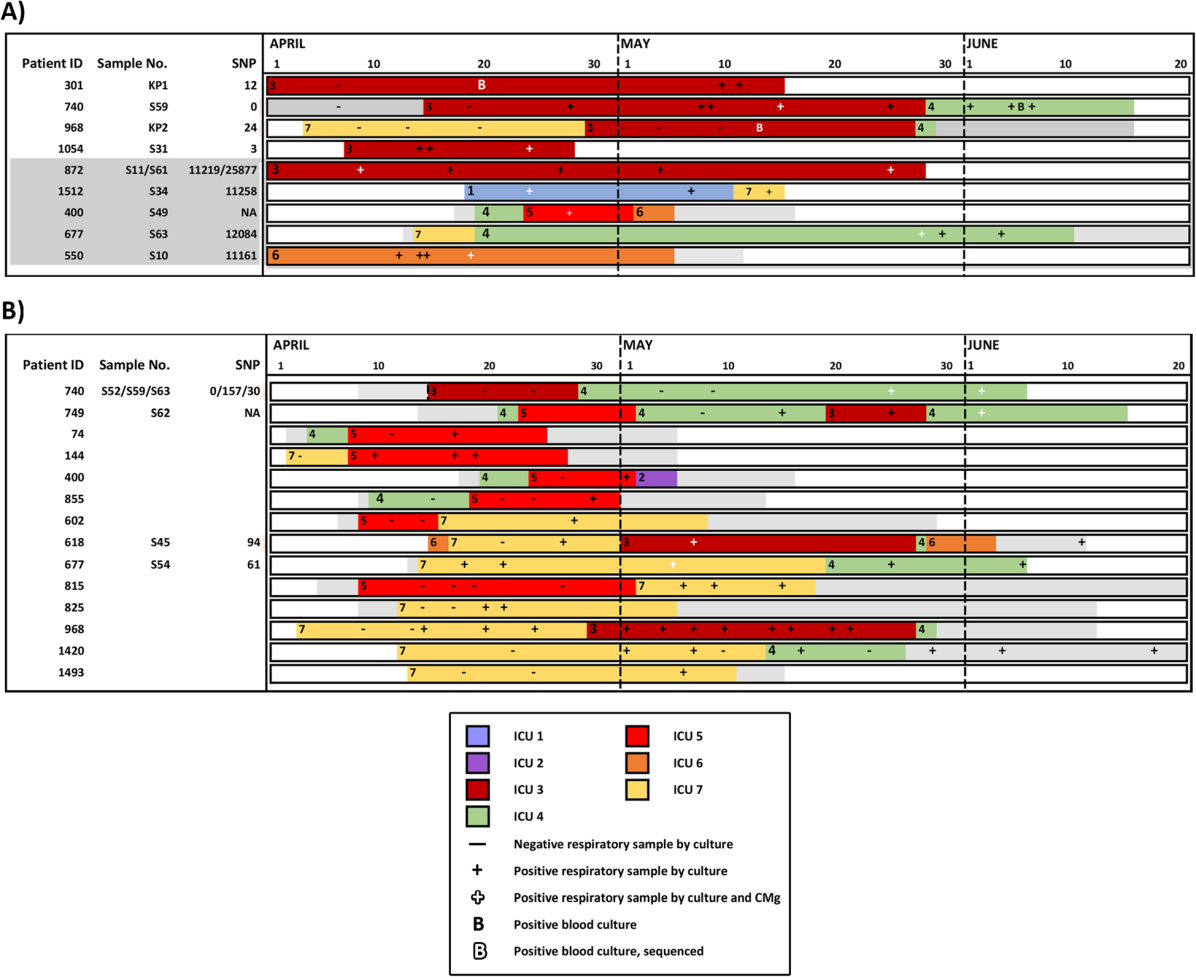
Identification of MDR *K. pneumoniae* and *C. striatum* outbreaks across the ICU network based on combined epidemiological and CMg analysis. Overlapping ward stays for patients involved in putative outbreaks of **A)** MDR *K. pneumoniae* and **B)** *C. striatum*. Each row represents a unique patient. Patients are ordered by ward of first positive (ascending) and then by patient ID (ascending). The horizontal axis shows the ward stays from April 1 to June 20. Non-ITU wards are coloured in grey. ITU wards are labelled 1–10 represented by a unique colour. Periods outside the hospital are represented in white. MDR-*K. pneumoniae* or *C. striatum* positive and negative respiratory samples by culture are marked as (+) or (-), respectively. Additionally, positive respiratory samples by CMg and culture are marked as “”. Positive blood cultures are marked as “**B**” and sequenced blood cultures are marked as “”. Patients with a CMg-aligned sequence have an S number (respiratory sample) or KP number (blood culture) adjacent to their identification number on the left of each bar. The number of SNPs for each CMg sample is also shown on the vertical axis. **A)** CMg was performed on MDR-*K. pneumoniae* in respiratory samples from patients 1054 and 301 and bloodstream infection isolates on patients 301 and 968 retrieved from the routine diagnostic laboratory (time point marked as “B”). Possible chain of transmission is from top to bottom. No sequenced patient could link 968 to 1517 or 618 to 740 and so were assumed to be due to cryptic transmission via other non-sequenced patients. **B)** CMg was performed on *C. striatum* in respiratory samples from patients 618, 677, 740 and 749. All other patients were linked by epidemiology only

The higher than anticipated prevalence of *Klebsiella* spp. and *C. striatum* in respiratory specimens raised the possibility of patient-to-patient transmission (Fig. 2). This was investigated by comparing genomes from all patients reported with these organisms identified by both culture and CMg, using reads obtained after 24 h of nanopore sequencing. Additional analysis combined with epidemiology linkage was then used to identify putative transmission networks amongst patients.

#### Klebsiella pneumoniae

Consensus sequence was generated using *K. pneumoniae* reads from 8 samples (8 patients). Different sequence types (ST) were determined in four samples (S11, S34, S59 and S63). No ST could be determined for three samples (S10, S31 and S61), and S49 was excluded from the analysis due to 3% genome coverage recovered (Additional file 1: Table S7A). Comparison of high-quality allele calls and pairwise comparison of bases from all 8 samples showed S31 was similar to S59 (ST307) with 55 SNP-based differences from 4,892,921 bases (99.999% identical). This indicates a recent evolutionary history with differences likely due to nanopore sequencing errors. All other samples differed by tens of thousands of SNPs (Additional file 1: Table S7B).

Two additional patients (301 and 968) had a *K. pneumoniae* bloodstream infection (BSI) with identical broad resistance phenotype as CMg samples S31 and S59 (patient 1054 and 740, respectively). Pairwise comparison of SNP differences across all 4 genomes showed they were virtually identical with 5–55 SNP differences (Additional file 1: Table S7C). Together with the epidemiological analysis (Fig. 2A), this supported the transmission of this *K. pneumoniae* ST307 clone between 4 patients implicating an unsuspected outbreak.

#### Klebsiella aerogenes

Consensus sequence generated using *K. aerogenes* reads from S1, S52 and S62 identified 49,007 SNPs from 4,647,134 bases in S1 and S52 (S62 was excluded due to low (1.5%) genome coverage) making them only 98.94% identical. Additionally, S1 and S52 had 3 alleles in common (pryG(3), rplB(1), rpoB(2)) but differed in the allele *leuS* (14 vs 29) indicating they had different sequencing types suggesting they were not part of an outbreak (remaining typing alleles were not fully called, and ST could not be determined).

#### Corynebacterium striatum

Analysis of consensus sequence using *C. striatum* reads from 5/6 samples (S45, S52, S54, S59 and S63—Additional file 1: Table S7D) from 4 patients showed 71,339 of 2,758,551 bases present in all consensus sequences (S62, patient 749, was excluded due to low (3.2%) genome coverage). Reviewing all positions where there was a base in all samples, the maximum distance was 157 SNPs from 1,486,708 bases (99.99% identity) implying they were part of an outbreak (Additional file 1: Table S7D). Epidemiological analysis of all 18 patients with *C. striatum* identified overlapping ward stays for 14/18 patients across three ICUs, with genome sequence data (from CMg samples) implicating an extensive outbreak associated with patient movement between ICUs (Fig. 2B).

## Discussion

COVID-19 ICUs are challenged with high rates of secondary infection and antimicrobial resistance, hence providing an impetus for the introduction of rapid (same day) results that can improve empiric treatment decisions. The current ‘gold standard’ culture-based diagnostics take > 48 h for pathogen and AMR identification [36]. CMg sequencing has the potential to provide same-day diagnosis (8 h turnaround) including pathogen identification [9, 12, 37–42] and antimicrobial resistance prediction [13, 25, 43]. This data can be used to provide targeted antimicrobial therapy, before the second dose of broad-spectrum antibiotics is administered, and to characterise outbreaks [13, 44, 45]. In our study, we illustrate the potential use of rapid CMg sequencing in COVID-19 ICU patients for improving antimicrobial stewardship and infection control investigations [46, 47]. A single respiratory CMg test provided bacterial and fungal identification and accurate AMR prediction within an 8-h laboratory workflow and data for molecular typing the following day (Fig. 1). Previous studies have provided examples of how metagenomics could be used for rapid diagnosis of infection and/or identifying transmission patterns, but here, these components are brought together with the background of expanded ICUs during the COVID-19 pandemic. Real-time provision of such data has the potential to fundamentally change the multi-disciplinary team approach to antimicrobial treatment, outbreak detection and AMR control on ICU.

CMg was 92% sensitive and 82% specific for bacterial and fungal detection, consistent with previous estimates [9, 16, 48] using pre-defined thresholds. Thresholds and rules added for pathogen identification were to remove low-quality reads, low-level reagent/laboratory contamination, bioinformatic misclassification of reads and/or barcode cross-talk [9]. Less stringent thresholds for fungal identification were used as *Aspergillus* can be present in very low numbers in respiratory samples, and any growth (even a single colony) on fungal culture plates is reported as significant (scanty growth was reported for all *Aspergillus* culture-positive samples). More sensitive thresholds for fungal detection was also implemented by other CMg studies [12]. Using our CMg test, only two pathogens were missed in 2 culture-positive samples. Missed pathogens were within polymicrobial samples reported as scanty growth and were detected in the samples by CMg, but below positivity thresholds. This indicates that the relative concentration of the missed pathogens was too low compared to the competing pathogens/bacteria in the samples to generate sufficient reads to pass thresholds. The clinical significance of minority pathogens in such samples could be questioned.

CMg also reported pathogens (*n* = 9) not identified by culture. Four out of the nine pathogens were reported in additional samples taken from these patients. From the remaining five pathogens, only two, *S. aureus* in S16 and S28, were likely to be false positive, probably due to *k-*mer misclassification of closely related non-pathogenic *Staphylococci* spp*.* (> 15,000 reads of *S. epidermidis* were reported in both samples). The remaining three organisms identified by metagenomics only, were likely to be true positives, as they are commonly found in respiratory samples, they were present at reasonable proportions of the reads and there was no evidence of cross-talk [9]. These pathogens could have been missed by culture because (a) the patients had received antibiotics prior to sampling, (b) they were present in samples with mixed infections and were not easily identified (Gram-negatives were reported in S37 and S44), or (c) the pathogen was present below the limit of detection of culture but not below the CMg LoD. Culture is a recognised imperfect gold standard, meaning the specificity of CMg is likely to have been underestimated.

We assessed how the impact of 2-h CMg AMR results could have modified the guideline-recommended empiric prescribing of piperacillin-tazobactam therapy, which is commonly used in the UK [49]. CMg accurately detected β-lactam resistance genes, consistent with phenotypic resistance to recommended antibiotics for the main respiratory pathogens, particularly *Enterobacterales*. Mismatch was only identified in 1 of 20 samples where an ESBL gene was identified in a sample containing phenotypically susceptible *K. pneumoniae*. CMg results would not inform piperacillin-tazobactam use in any case highlighting the shortcomings of making a single empiric antibiotic recommendation when such a broad range of bacteria and resistance phenotypes are possible. We could not compare carbapenem resistance with carriage of carbapenemase genes in *Enterobacterales* because neither were detected in this cohort. However, this is expected to be feasible using CMg sequencing as demonstrated previously by other studies [9, 50]. Also, we did not attempt to determine phenotype from mutational resistances due to nanopore sequencing errors or from plasmid-borne resistances, as due to plasmid promiscuity would be challenging to determine the plasmid’s host. However, both challenges could be overcome by using CMg data for genomic neighbour typing as previously demonstrated [13].

CMg also demonstrated potential for accurately diagnosing IPA. It detected all culture-positive samples and was 100% concordant with targeted qPCR, whereas half the patients with a positive GM result were not confirmed by the other three testing methodologies. Diagnosing secondary IPA is difficult with severe viral infections [4] and particularly COVID-19 patients, who commonly fulfil all radiological, clinical and host diagnostic criteria [51]. IPA in COVID-19 patients was uncommon in our study (about 2%) as in other London centres [52]. The single small focus of the IPA in only one post-mortem reported here and elsewhere [53] suggests COVID-19-related IPA may not be as clinically significant as with influenza; however, this study was done during the first wave prior to evidence for benefit of steroid and toculizimab treatment that might increase frequency and severity of IPA. These encouraging preliminary CMg performance metrics need follow-up with larger sample cohorts to assess this technologies’ potential as a diagnostic tool for IPA.

Finally, using 24-h CMg data, we identified the contribution of transmission towards the high prevalence of *Klebsiella* spp. and *C. striatum* observed here and elsewhere [54]. CMg identified an MDR-*K. pneumoniae* ST307 outbreak which is a particular concern given its resistance profile and extensive international spread [55]. CMg also identified an MDR-*C. striatum* outbreak potentially involving 14 patients. The clinical significance of detecting *C. striatum* in respiratory specimens is unclear although MDR-*C. striatum* outbreaks have been reported [56]. These findings highlight again the benefit of unbiased pathogen detection using CMg in revealing hidden outbreaks.

Further work is now required to consider CMg as a clinical service. For example, samples were batched in this study (6 per run) whereas delaying sequencing of specimens for batching reduces the benefit of having a rapid test. Singleplex sequencing using Flongle flow cells would be suitable for single runs, but processing samples at different times would have a significant impact on the microbiology laboratory workflow. Another issue is that only a small proportion of total COVID-19 ICU patients and samples were tested over 9 weeks due to limited staffing resources. For routine service, a scale up of resources would be required or decisions would need to be made on sample prioritisation.

The current method can only detect bacteria and fungi but not viruses. Modifying sample preparation could allow viral detection, enabling parallel diagnosis of respiratory infections independent of the causative agent. The negative control rule applied in this study was implemented to remove reagent or laboratory contaminants, such as *E. coli*, as previously done in other CMg studies [57, 58]*.* However, removing contaminants in this way could result in the removal of true pathogens (such as *E. coli*) from respiratory samples which could negatively impact the sensitivity (not the case in this study). Alternatively, removing a certain proportion of contaminant reads from samples on the run could be done instead [59]. CMg-only dedicated laboratories with strict aseptic contamination-free techniques should be used for sample handling for CMg. Also, an internal process control (IPC) could be used to tell the difference between method failures and true-negative samples. Further work is required to improve the bioinformatics analysis to minimise misclassification of closely related species. Both the development of the IPC and improved bioinformatics are underway.

## Conclusions

This study demonstrates the potential for a single rapid CMg test to improve treatment of bacterial and fungal infections, improve antimicrobial stewardship and help identify nosocomial transmission and target infection control interventions. It demonstrates the full benefit of CMg for the whole multi-disciplinary team across laboratory scientists, intensivists, pharmacists and infection control experts, particularly in an ICU setting during this COVID-19 pandemic where the capacity challenges and disease severity can create unpredictable epidemiology and high levels of AMR [60]. The provision of such evidence for these hospital professional groups is required to get engagement on moving away from a predominantly culture-based approach and justify investment in CMg. Further clinical evaluation of an ICU CMg service is our priority as this COVID-19 pandemic continues.

## Supplementary Information

**Additional file 1: Supplementary Methods and Tables S1-S8**. **Table S1.** List of pre-defined pathogen and reference source for each pathogen. **Table S2.** All non-pathogenic organisms identified in all respiratory samples processed with clinical metagenomics (above pre-defined thresholds). **Table S3.** Clinical characteristics and results of routine microbiological tests performed on intubated COVID-19 patients across 7 linked dedicated COVID-19 intensive care units on Guy’s and St Thomas’ hospital sites during the first wave of the COVID-19 pandemic. **Table S4A.** Sequencing metadata for all respiratory samples processed with clinical metagenomics. **Table S4B.** Negative controls run with each batch of samples sequenced. **Table S5.** Phenotypic resistance reported by culture and resistance genes reported by clinical metagenomics in all culture-positive samples after 2 hours of sequencing. **Table S6.** Microbiology, PCR and clinical metagenomics results for all samples processed in this study for the identification of *Aspergillus fumigatus*. **Table S7A-D.** *Klebsiella pneumoniae* and *Corynebacterium striatum* alignment for outbreak analysis. **Table S8A-E.** Performance reported after testing different parameters on training set for pathogen identification. The number of True Positive (TP), False Positive (FP), True Negative and False Negative (TN) samples as well as sensitivity, specificity along and calculated Youden’s Index ((sensitivity+specificity)-1) are presented.

**Additional file 2: Figs. S1-S3**. **Fig. S1A-B.** Post mortem histological analysis of focal invasive pulmonary aspergillosis (IPA). **Fig. S2.** Receiver Operator Curve (ROC) curve analysis based on discordant testing (CMg+qPCR) performed for the training set. **Fig. S3.** WIMP alignment q-score plotted against the equivalent centrifuge score. Tested WIMP alignment q-scores are plotted on the y axis against the equivalent centrifuge score on the x axis.

## Data Availability

Sequence data presented in this study are available on the European Nucleotide Archive (ENA) under project number PRJEB41184 (https://www.ebi.ac.uk/ena/browser/view/PRJEB41184?show=reads [61]).

## Abbreviations

AMR: Antimicrobial resistance
BAL: Bronchoalveolar lavage
BSI: Bloodstream infections
CMg: Clinical metagenomics
FAA: Fastidious anaerobic agar
GM: Galactomannan
GNB: Gram-negative bacteria
ICU: Intensive care unit
IPA: Invasive pulmonary aspergillosis
IPC: Internal positive control
LoD: Limit of detection
NDL: Non-direct bronchoalveloar lavage
PPE: Personal protective equipment
ST: Sequence type
VAP: Ventilator-associated pneumonia
