# Supplementary material for: Evaluating the potential for respiratory metagenomics to improve treatment of secondary infection and detection of nosocomial transmission on expanded COVID-19 intensive care units
Source: Genome Med. 2021 Nov 17;13:182. doi: 10.1186/s13073-021-00991-y (PMC8594956; doi:10.1186/s13073-021-00991-y)
Supplement: Supplementary file 1 — Additional file 1: Supplementary Methods and Tables S1-S8. Table S1. List of pre-defined pathogen and reference source for each pathogen. Table S2. All non-pathogenic organisms identified in all respiratory samples processed with clinical metagenomics (above pre-defined thresholds). Table S3. Clinical characteristics and results of routine microbiological tests performed on intubated COVID-19 patients across 7 linked dedicated COVID-19 intensive care units on Guy’s and St Thomas’ hospital sites during the first wave of the COVID-19 pandemic. Table S4A. Sequencing metadata for all respiratory samples processed with clinical metagenomics. Table S4B. Negative controls run with each batch of samples sequenced. Table S5. Phenotypic resistance reported by culture and resistance genes reported by clinical metagenomics in all culture-positive samples after 2 hours of sequencing. Table S6. Microbiology, PCR and clinical metagenomics results for all samples processed in this study for the identification of Aspergillus fumigatus. Table S7A-D. Klebsiella pneumoniae and Corynebacterium striatum alignment for outbreak analysis. Table S8A-E. Performance reported after testing different parameters on training set for pathogen identification. The number of True Positive (TP), False Positive (FP), True Negative and False Negative (TN) samples as well as sensitivity, specificity along and calculated Youden’s Index ((sensitivity+specificity)-1) are presented. [file 13073_2021_991_MOESM1_ESM.docx]

**Supplementary Material**

**Evaluating the potential for respiratory metagenomics to improve treatment of secondary infection and detection of nosocomial transmission on expanded COVID-19 intensive care units**

Themoula Charalampous^1^*, Adela Alcolea-Medina^1,2^*, Luke B. Snell^1,3^*, Tom G.S. Williams^3^, Rahul Batra^1,3^, Christopher Alder ^1,3^, Andrea Telatin^6^, Luigi Camporota^4^, Christopher I.S. Meadows^4^, Duncan Wyncoll^4^, Nicholas A. Barrett^4^, Carolyn J. Hemsley^3^, Lisa Bryan^2^, William Newsholme^3^, Sara E. Boyd^3^, Anna Green^5^, Ula Mahadeva^5^, Amita Patel^1,3^, Penelope R. Cliff^2^, Andrew J. Page^6^, Justin O’Grady^6^^ and Jonathan D. Edgeworth^1,2,3^^.

^1^ Centre for Clinical Infection and Diagnostics Research, Department of Infectious Diseases, School of Immunology and Microbial Sciences, Kings College London, London

^2^ Infection Sciences, St Thomas’ Hospital, Viapath, London

^3^ Department of Infectious Diseases, Guy’s and St Thomas’ Hospital NHS Foundation Trust, London

^4^ Critical Care Directorate, Guy’s and St Thomas’ Hospital NHS Foundation Trust, London

^5^Department of Cellular Pathology, Guy's and St Thomas' NHS Foundation Trust, London, UK.

^6^ Quadram Institute Bioscience, Norwich Research Park, Norwich, UK

*These authors contributed equally

^Senior and corresponding authors

# **Supplementary Methods**

## **Determining parameters for pathogen Identification in respiratory samples**

### Training dataset and definition of thresholds

Parameters used in this study were chosen prior to sequencing data analysis using the dataset published by Charalampous *et al* (2019) (1) as the training set to identify the best parameters. Different centrifuge scores and percentage microbial classified reads thresholds were tested. Rules were also applied to remove barcode leaking and contamination. The training set is consisted of 41 respiratory metagenomic samples and 7 processed extraction controls. The CMg+qPCR results from the paper were used as the gold standard ‘true dataset’ (1).

Sensitivity was calculated per sample (rather than per pathogen) basis and if all pathogen/s reported by CMg+qPCR were identified then sample was considered as ‘true-positive’. Additional detections by metagenomics in true-positive samples were not considered false-positive. Specificity was also calculated on a per sample basis. Additional detections in negative sample were considered as false-positive. Reads for all pathogens present on the pre-defined list were reported after thresholds were applied. If confirmatory testing for any identified pathogen was not available, then original culture result was considered to be correct. Youden’s Index ((sensitivity+specificity)-1)) was calculated for each tested threshold. Also, ROC curves of the true-positive rate against the false-positive rate were generated for the different thresholds tested.

Previous knowledge from metagenomic sequencing of respiratory specimens highlighted that thresholds are necessary to identify and remove low-quality reads, contamination introduced during sample processing, bioinformatic misclassification during analysis and barcode cross-talk (1). Hence, different percentages of microbial classified reads (≥: 0.1%, 1%, 2%, 5%, 10% 15%) and centrifuge scores (≥ 338, 1023, 2504, 5838, 13420) were tested to determine the best parameters for identifying pathogens whilst avoiding false-positives. The q-score is no longer an adjustable in WIMP (removed by Oxford Nanopore Technologies (ONT)). The WIMP q-scores were derived from the centrifuge scores hence we determined the centrifuge scores equivalent to WIMP q-scores 10, 15, 20, 25 and 30 and tested (Additional file [2](https://figshare.com/articles/figure/Additional_file_2/16685188): Figure S2).

Centrifuge score ≥13420 and 0.1% microbial classified reads gave the highest Youden’s index (78%) with 94% sensitivity and 83% specificity (Table S8). However, the highest true-positive percentage (100%) was reported with centrifuge score of ≥2504 and ≥1% microbial classified reads (Additional file [2](https://figshare.com/articles/figure/Additional_file_2/16685188): Figure S3) with a Youden’s index of 67%. We chose these thresholds to maximise sensitivity and minimise false negative results (major errors).

Additional rules were also applied to allow identification of barcode cross-talk and exclude potential contaminants. Barcode cross-talk reads in extraction controls were present at 0.01-0.06% of the classified pathogen reads. This typically became an issue when >10,000 reads were reported for a particular pathogen in a run (cumulative from all barcoded samples on the run). Hence, to ensure barcode cross-talk didn’t result in false positive results, we removed 0.1% of pathogen reads from all samples (i.e. from each barcode) if there were >10,000 cumulative reads for a single pathogen identified from the 6 samples on the flowcell. Any pathogen reads remained in extraction negative (with >5 classified reads) were considered contaminants and were the pathogen/s were removed from the analysis on the run. These rules were applied when determining the best Centrifuge and pathogen read thresholds using ROC curves.

### Determining *Aspergillus* specific threshold

The clinical microbiology laboratory tests for *Aspergillus* infection in COVID-19 patients using Galactomannan antigen testing in serum and BAL and culture on fungal-selective media (Saburaud Dextrose Agar (SAB)). A sample is considered positive for *Aspergillus­* if a single colony is observed which is more stringent than the thresholds used for microbial culture (estimated culture LoD for bacteria is ~10^2^ cfu/ml – i.e. one colony on plate streaked with an immersed swab from an undiluted sample). A more sensitive species-specific threshold was therefore required for *Aspergillus* detection. A threshold of ≥10 *Aspergillus* reads was used to identify a sample positive for Aspergillus. This was the lowest threshold we could comfortably choose that would provide the highest sensitivity while avoiding false positive detections. This threshold needs further validation as we could not use the training set to choose the best threshold (no *Aspergillus* detected in that dataset).

**Table S1.** List of pre-defined pathogen and reference source for each pathogen.

| **Bacteria** | **Reference** |
| --- | --- |
| *Acinetobacter baumannii* complex | (2-4) |
| *Burkholderia spp.* | (3) |
| *Citrobacter koseri* | (2, 4) |
| *Citrobacter freundii* | (2, 4) |
| *Enterobacter aerogenes* | (1-4) |
| *Escherichia coli* | (1-4) |
| *Enterobacter cloacae* | (1-4) |
| *Haemophilus influenzae* | (1-3) |
| *Klebsiella pneumoniae* | (1-4) |
| *Klebsiella oxytoca* | (1-4) |
| *Klebsiella variicola* | (2-4) |
| *Legionella pneumophila* | (2-4) |
| *Moraxella catarrhalis* | (1-4) |
| *Morganella morganii* | (2-4) |
| *Pseudomonas aeruginosa* | (1-4) |
| *Proteus mirabilis* | (1-4) |
| *Staphylococcus aureus* | (1-4) |
| *Serratia marcescens* | (1-4) |
| *Streptococcus pneumoniae* | (1-3, 5) |
| *Stenotrophomonas maltophilia* | (1, 3, 4) |
| *Streptococcus agalactiae* | (2-4) |
| *Streptococcus pneumoniae* | (1-5) |
| *Streptococcus pyogenes* | (1-4) |
| **Fungi** |  |
| *Aspergillus spp.* | (3) |

**Table S2.** All non-pathogenic organisms^a^ identified in all respiratory samples processed with clinical metagenomics (above pre-defined thresholds^b^).

| **Sample ID** | **Non-pathogenic organisms identified above pre-defined thresholds by respiratory metagenomics** | **Classified reads of reported pathogens after chosen thresholds** |
| --- | --- | --- |
| S1 | N/A | - |
| S5 | N/A | - |
| S8 | *Cutibacterium acnes*  *Cutibacterium acnes* HL096PA1  *Moraxella osloensis* | 69  10  14 |
| S10 | *Prevotella denticola* F0289 | 7647 |
| S11 | *Candida dubliniensis Candida albicans* | 62  40 |
| S14 | N/A | - |
| S16 | *Candida ortholipsilosis*  *S. epidermidis*  *S. haemolyticus,*  *Lactobacillus paracasei*  *Lactobacillus casei* | 31  18036  11745  2308  1122 |
| S17 | N/A | - |
| S18 | *Candida glabrata*  *Candida dublinensis*  *Tannerella forsythia*  *Olsenella uli* | 6721  6082  2197  898 |
| S19 | *C. orthopsilosis* | 202 |
| S20 | N/A | - |
| S21 | *Candida albicans*  *Enterobacter hormaechei subsp. oharae,*  *Enterobacter hormaechei subsp. steigerwaltii* | 43  1118  5731 |
| S25 | N/A | - |
| S27 | *Cutibacterium acnes* HL096PA1  *Cutibacterium acnes* KPA171202  Arthrobacter sp. *IHBB 11108*  *Bacillus subtilis* BEST7003  *Moraxella osloensis* | 94  86  100  79  42 |
| S28 | *Candida albicans*  *E. faecium*  *Staphylococcus epidermidis*  *Staphylococcus epidermidis* ATCC 12228  *Staphylococcus haemolyticus* JCSC1435  *Staphylococcus haemolyticus* | 37  7464  16118  759  2219  1403 |
| S29 | *Cutibacterium acnes*  *Bacillus subtilis* BEST7003  *Arthrobacter sp.* IHBB 11108  *Moraxella osloensis*  *Mucilaginibacter sp.* PAMC 26640 | 22  21  19  11  7 |
| S30 | N/A | - |
| S31 | N/A | - |
| S33 | *Cutibacterium acnes*  *Atopobium parvulum* DSM 204699  *Arthrobacter sp*. IHBB 11108  *Bacillus subtilis* BEST7003  *Moraxella osloensis* | 39  13  29  26  13 |
| S34 | *Candida albicans*  *Streptococcus oralis*  *Streptococcus oralis* Uo5  *Prevotella melaninogenica*  *Prevotella sp. oral taxon 299 str.* F0039 | 3962  12638  1841  2117  1313 |
| S35 | *Acinetobacter pittii,*  *Acinetobacter johnsonii* XBB1  *Acinetobacter sp.* TTH0-4  *Acinetobacter schindleri*  *Acinetobacter sp*. NCu2D-2  Acinetobacter nosocomialis | *22*  *15*  *4*  *9*  *7*  *8* |
| S36 | N/A | - |
| S37 | *Neisseria sicca*  *Neisseria elongata subsp. glycolytica* ATCC 29315 | 10121  468 |
| S39 | N/A | - |
| S40 | N/A | - |
| S41 | *Candida albicans*  *Eikenella corrodens*  *Enterococcus faecium*  *Streptococcus constellatus subsp. pharyngis*  *Streptococcus anginosus* C238  *Streptococcus anginosus subsp. whileyi* MAS624  *Streptococcus intermedius* JTH08  *Staphylococcus epidermidis*  *Prevotella melaninogenica*  *Prevotella intermedia*  *Rothia dentocariosa* ATCC 17931 | 505  3243  4,016  1985  314  270  313  758  370  553  464 |
| S42 | *Burkholderia thailandensis*  *Burkholderia thailandensis* 2002721643  *Burkholderia pseudomallei* | 749  499  1478 |
| S44 | N/A | - |
| S45 | *Corynebacterium simulans*  *Corynebcaterium striatum*  *Corynebacterium resistens* DSM 45100 | 1395  8,665  141 |
| S46 | *Cutibacterium acnes* | 25 |
| S49 | *Candida albicans*  *Streptococcus sanguinis* SK36  *Bacillus subtilis* BEST7003  *Arthrobacter sp.* IHBB 11108  *Staphylococcus epidermidis*  *Enterococcus faecium*  *Cutibacterium acnes*  *Mucilaginibacter sp.* PAMC 26640 | 594  165  144  138  41  42  32  28 |
| S51 | N/A | - |
| S52 | *Corynebacterium simulans*  *Corynebacterium striatum*  *Candida albicans* | *3795*  24,347  *78* |
| S53 | N/A |  |
| S54 | *Corynebacterium simulans,*  *Corynebacterium striatum*  *Corynebacterium resistens* DSM 45100  *Cutibacterium acnes*  *Corynebacterium aurimucosum* ATCC 700975 | *280*  1758  *28*  *50*  *28* |
| S55 | *Sphingomonas sp.* LK11 | 418 |
| S56 | *E. faecium* | 19568 |
| S59 | *Corynebacterium simulans*  *Corynebacterium striatum* | 1393  8478 |
| S61 | N/A | - |
| S62 | *Corynebacterium simulans*  *Corynebacterium striatum*  *Arthrobacter sp.* IHBB 11108  *Bacillus subtilis* BEST7003  *Staphylococcus epidermidis*  *Mucilaginibacter sp*. PAMC 26640  *Corynebacterium kroppenstedtii* DSM 44385  *Cutibacterium acnes* | 42  227  41  41  15  17  14  9 |
| S63 | *Corynebacterium simulans*  *Corynebacterium striatum*  *Corynebacterium aurimucosum* ATCC 700975  *Corynebacterium resistens* DSM 45100 | 13535  89166  1419  1389 |
| S64 | *Candida albicans*  *Prevotella melaninogenica,*  *Streptococcus gordonii,*  *Streptococcus gordonii str. challis substr.* CH1  *Actinomyces pacaensis*  *Prevotella intermedia*  *Fusobacterium nucleatum subsp. vincentii*  *Prevotella fusca* JCM 17724,  *Parabacteroides sp.* CT06,  *Porphyromonas gingivalis* | 39  7671  1497  383  1448  845  245  250  390  315 |
| S65 | *Candida albicans*  *Prevotella melaninogenica*  *Atopobium parvulum* DSM 20469,  *Streptococcus oralis*  *Streptococcus oralis subsp. tigurinus*  *Prevotella intermedia*  *Fusobacterium nucleatum subsp. vincentii,*  *Fusobacterium nucleatum subsp. vincentii* 3_1_36A2  *Streptococcus gordonii*  *Porphyromonas gingivalis*  *Parabacteroides sp. CT06* | 30  9164  1885  834  1217  1655  397  377  721  746  599 |

^a^pre-defined thresholds are: ≥1%microbial classified, ≥2504 alignment score and barcode cross-talk rule. ^b^Not defined as pathogenic organisms in this study

**Table S3**. Clinical characteristics and results of routine microbiological tests performed on intubated COVID-19 patients across 7 linked dedicated COVID-19 intensive care units on Guy’s and St Thomas’ hospital sites during the first wave of the COVID-19 pandemic

|  | **All**  **(n = 274)** | **Metagenomics group^a^**  **(n = 34)** | **Non-metagenomics group**  **(n = 240)** |
| --- | --- | --- | --- |
| Median age (IQR) | 56 (45-63) | 52 (41-58) | 56 (46-63) |
| Sex – Male | 195 (71%) | 23 (70%) | 172 (72%) |
| Ethnicity  White  Black and Minority Ethnicities  Not known | 99 (36%)  110 (40%)  65 (24%) | 16(47%)  15 (44%)  3 (9%) | 83 (34%)  95 (40%)  62 (26%) |
| Mortality | 78 (29%) | 8 (24%) | 71 (30%) |
| Length of stay (IQR) | 19 days (12-37) | 32 days (24-47) | 17 days (11-32) |
| **Respiratory Cultures in ITU** | | | |
| Median samples per patient (IQR) | 2 (1 – 4) | 4 (4 – 6) | 2 (1 – 3) |
| Total number of samples / Patients tested | 763 / 226 | 180 / 34 | 580 / 192 |
| **Organisms from respiratory culture whilst in ICU (Number of individuals who ever had the following organisms in any sample)** | | | |
| *Klebsiella spp+.* | 86 (31%) | 18 (53%) | 67 (28%) |
| *Staphylococcus aureus* | 27 (10%) | 3 (9%) | 24 (10%) |
| *Citrobacter spp.* | 23 (8%) | 5 (15%) | 17 (7%) |
| *Escherichia coli* | 19 (7%) | 3 (9%) | 15 (6%) |
| *Pseudomonas spp.* | 19 (7%) | 1 (3%) | 17 (7%) |
| *Corynebacterium striatum* | 18 (7%) | 8 (24%) | 10 (4%) |
| *Enterococcus spp.* | 18 (7%) | 4 (12%) | 14 (6%) |
| *Serratia spp.* | 14 (5%) | 2 (6%) | 12 (5%) |
| *Enterobacter spp.* | 10 (4%) | 1 (3%) | 9 (4%) |
| *Haemophilus spp.* | 8 (3%) | 0 (0%) | 6 (3%) |
| *Stenotrophomonas maltophilia* | 7 (3%) | 1 (3%) | 5 (2%) |
| *Proteus spp.* | 4 (1%) | 4 (12%) | 0 (0%) |
| *Morganella spp.* | 3 (11%) | 1 (3%) | 2 (1%) |
| *Acinetobacter spp.* | 2 (1%) | 1 (3%) | 1 (0%) |
| *Streptococcus pyogenes* | 1 (0.3%) | 0 (0%) | 1 (0%) |
|  |  |  |  |
| *Candida albicans* | 76 (28%) | 13 (38%) | 64 (27%) |
| *Candida spp.* (non *albicans*) | 21 (8%) | 5 (15%) | 15 (6%) |
| *Aspergillus spp.* | 6 (2%) | 3 (9%) | 1 (0%) |
|  |  |  |  |
| No organisms isolated | 74 (27%) | 2 (6%) | 73 (30%) |
| **Galactomannans (GAL)** | | | |
| **Bronchoalveolar lavage (BAL) GALs** | | | |
| Number of tests / Patients tested | 76 / 51 | 25 / 16 | 48 / 33 |
| Positive tests / Patients positive | 10 / 8 | 6 / 5 | 1 / 1 |
| **Serum GALs** | | | |
| Number of tests / Patients tested | 119 / 74 | 34 / 22 | 84 / 52 |
| Positive tests / Patients positive | 7 / 7 | 3 / 3 | 4 / 4 |

^a^1 patient was SARS-CoV-2 RNA PCR negative but had clinical diagnosis of COVID-19.

| **Table S4A.** Sequencing metadata for all respiratory samples processed with clinical metagenomics. | | | | | | | |
| --- | --- | --- | --- | --- | --- | --- | --- |
| **Sample ID** | **Human**  **reads** | **Microbial classified reads** | **Unclassified reads** | **Microbial reads** | **Number of raw reads from 2hrs** | **Metagenomics output** | **Classified reads of reported pathogens after chosen thresholds**^a^ |
| S1^N3^ | 2,805 | 144,220 | 975 | 145195 | 148,000 | *K. aerogenes* | 138,626 |
| S5^N3^ | 16 | 0 | 0 | 0 | 16 | Negative | 0 |
| S8^N5^ | 736 | 579 | 820 | 1,399 | 2135 | *A. fumigatus K. oxytoca* | 77  44 |
| S10^N5^ | 4,118 | 74,891 | 459 | 75,350 | 79,468 | *K. pneumoniae* | 69,029 |
| S11^N5^ | 39,675 | 28,473 | 7,195 | 35,668 | 68,148 | *K. pneumoniae* | 16,828 |
| S14^N6^ | 3,795 | 195 | 10 | 205 | 4000 | Negative | 0 |
| S16^N6^ | 8,308 | 42,269 | 1,423 | 43,692 | 52,000 | *S. aureus* | 1,768 |
| S17^N7^ | 6,095 | 1790 | 115 | 1,905 | 8000 | *P. aeruginosa* | 1,457 |
| S18^N6^ | 17,224 | 19,478 | 3,298 | 22,776 | 40,000 | Negative | 0 |
| S19^N6^ | 37,629 | 6,866 | 3,505 | 10,371 | 48,000 | Negative | 0 |
| S20^N7^ | 21,292 | 38,361 | 347 | 38,708 | 60,000 | *S. aureus* | 36,281 |
| S21^N6^ | 134 | 75,112 | 754 | 75,866 | 76000 | *E. cloacae* | 62,314 |
| S25^N15^ | 62,587 | 1325 | 88 | 1,413 | 64,000 | Negative | 0 |
| S27^N11^ | 11,214 | 4,238 | 548 | 4,786 | 16,000 | Negative | 0 |
| S28^N11^ | 87,316 | 43,293 | 1,391 | 44,684 | 132000 | *S. aureus A. fumigatus* | 3,165  2,649 |
| S29^N10^ | 1108 | 439 | 320 | 759 | 1867 | Negative | 0 |
| S30^N10^ | 200 | 91 | 136 | 227 | 307 | Negative | 0 |
| S31^N10^ | 200 | 31,233 | 567 | 31,800 | 32,000 | *K. pneumoniae* | 28,056 |
| S33^N10^ | 16,179 | 981 | 195 | 1,176 | 17,355 | Negative | 0 |
| S34^N10^ | 27,481 | 76,803 | 5,993 | 82,796 | 110,277 | *K. pneumoniae* | 38,758 |
| S35^N10^ | 18 | 465 | 388 | 853 | 871 | *A. baumanni* | 99 |
| S36^N8^ | 119 | 117,999 | 1,882 | 119,881 | 120,000 | *S. aureus* | 109,767 |
| S37^N8^ | 10,605 | 31,895 | 13,500 | 45,395 | 56,000 | *M morganni K. pneumoniae P. mirabilis* | 28,300  876  397 |
| S39^N8^ | 1,749 | 25804 | 447 | 26251 | 28,000 | *C. koseri K. pneumoniae* | 23,870  284 |
| S40^N11^ | 78 | 1024 | 133 | 1157 | 1235 | Negative | 0 |
| S41^N8^ | 933 | 24,770 | 6,297 | 31,067 | 32,000 | *S. aureus* | 1,365 |
| S42^N8^ | 449 | 45474 | 6,077 | 51551 | 52,000 | *Burkholderia spp.* | 34,347 |
| S44^N13^ | 2,922 | 63,370 | 1,708 | 65,078 | 68,000 | *S. marcesens C. freundi K. aerogenes^b^* | 53,082  6,082 173 |
| S45^N16^ | 12,239 | 13,256 | 2,505 | 15,761 | 28,000 | *K. aerogenes^b^* | 104 |
| S46^N13^ | 766 | 300 | 162 | 462 | 1,228 | *C. koseri* | 237 |
| S49^N13^ | 33,798 | 2,070 | 132 | 2,202 | 36,000 | *K. pneumoniae* | 594 |
| S51^N14^ | 87,418 | 8,342 | 240 | 8,582 | 96,000 | *S. aureus C. koseri* | 5,203  2,262 |
| S52^N7^ | 49,146 | 37,875 | 979 | 38,854 | 88,000 | *K. aerogenes* | 5,277 |
| S53^N15^ | 24 | 0 | 0 | 0 | 24 | Negative | 0 |
| S54^N14^ | 8,985 | 2,698 | 317 | 3,015 | 12,000 | Negative | 0 |
| S55^N11^ | 26,854 | 8,724 | 422 | 9,146 | 36,000 | *A. fumigatus* | 16 |
| S56^N11^ | 3,342 | 36,278 | 380 | 36,658 | 40,000 | *P. mirabilis A. fumigatus* | 11,323  79 |
| S59^N14^ | 542 | 117,780 | 1,678 | 119,458 | 120,000 | *K. pneumoniae* | 99,186 |
| S61^N15^ | 76 | 47,159 | 765 | 47,924 | 48,000 | *C. koseri K. pneumoniae P. mirabilis* | 29,797  14,118  815 |
| S62^N14^ | 10,979 | 920 | 101 | 1,021 | 12,000 | *K. aerogenes* | 184 |
| S63^N15^ | 621 | 141,567 | 5,812 | 147,379 | 148,000 | *K. pneumoniae* | 17,0341 |
| S64^N16^ | 3,516 | 19,652 | 4,832 | 24,484 | 28,000 | Negative | 0 |
| S65^N16^ | 26,010 | 32,859 | 13,131 | 45,990 | 72,000 | Negative | 0 |

^a^Pre-defined thresholds: ≥1%microbial classified, >2503 alignment score and barcode cross-talk rule applied.

^b^Pathogens identified but were not above pre-defined thresholds

**Table S4B**. Negative controls run with each batch of samples sequenced*.

| **Control ID** | **Human reads** | **Microbial classified reads** | **Unclassified reads** | **Number**  **of**  **reads**  **minus**  **hg38** | **Number of raw reads**  **by**  **CMG** | **Pathogen reported**  **by**  **CMg** | **Pathogen classified reads**  **by**  **CMg** | **Contaminant**  **reported**  **by**  **CMg** |
| --- | --- | --- | --- | --- | --- | --- | --- | --- |
| N3 | 0 | 35 | 0 | 0 | 35 | *K. aerogenes*  *M. catarrhalis P. aeruginosa* | 23  6  5 | *M. catarrhalis P. aeruginosa* |
| N5 | 2 | 136 | 112 | 248 | 250 | *E. coli*  *K. pneumoniae* | 9  4 | *E. coli* |
| N6 | 2946 | 898 | 156 | 1054 | 4,000 | *E. coli* | 76 | *E. coli* |
| N7 | 0 | 0 | 9 | 0 | 9 | - | 0 | *-* |
| N8 | 414 | 89 | 10 | 99 | 513 | *E. coli*  *S. aureus* | 9  4 | *E. coli* |
| N10 | 5812 | 1599 | 288 | 1887 | 7699 | *E. coli*  *K. pneumoniae* | 390  20 | *E. coli* |
| N11 | 238 | 1234 | 114 | 1348 | 1586 | *E. coli*  *H. influenzae*  *K. pneumoniae* | 290  14  11 | *E. coli*  *H. influenzae* |
| N13 | 41,567 | 2,420 | 13 | 2,433 | 44,000 | *E. coli* | 63 | *E. coli* |
| N14 | 15 | 402 | 63 | 465 | 480 | *E. coli*  *K. pneumoniae* | 37  15 | *E. coli* |
| N15 | 56 | 80 | 8642 | 6942 | 6998 | *E. coli*  *K. pneumoniae* | 13  9 | *E. coli* |
| N16 | 1248 | 480 | 0 | 480 | 1728 | - | 0 | - |

*after 2hrs of sequencing

**Table S5**. Phenotypic resistance reported by culture and resistance genes reported by clinical metagenomics in all culture-positive samples after 2 hours of sequencing.

| **Sample number** | **Pathogen identified by culture** | **Reported phenotypic resistance**^a^ | **AMR genes reported by metagenomic sequencing** |
| --- | --- | --- | --- |
| S1 | *K. aerogenes* | Amoxicillin  Co-amoxiclav  Cefuroxime  Ceftazidime  Pireracillin/Tazobactam | ND |
| S8 | *A. fumigatus* | ND | ND |
| S10 | *K. pneumoniae* | Amoxicillin | *oqcA_1 fosA_5* |
| S11 | *K. pneumoniae* | Amoxicillin | *oqxB_1 oqcA_1 fosA5_1 fosA_6*  *fosA_5*  *fosA_5 oqcA_1* |
| S17 | *P. aeruginosa* | Sensitive | ND |
| S20 | *S. aureus* | Penicillin  Erythromycin  Fusidic acid | *fusB_1*  *fosD_1*  *aadD_1*  *erm(T)_2* |
| S21 | *E. cloacae* | Ampicillin | *fosA_1*  *fosA_7* |
| S28 | *Aspergillus* | ND | ND |
| S31 | *K. pneumoniae* | ESBL  Ciprofloxacin  Penicillins  Septrin  Amoxicillin | *aph(6)-Id_1*  *oqxB_1*  *aph(3'')-Ib_5*  *blaOXA-1_1*  *dfrA14_1*  *oqxA_1*  *fosA6_1*  *sul2_2*  *blaTEM-1B_1*  *blaSHV-28_1*  *sul2_15*  *blaCTX-M-103_1* |
| S34 | *K. pneumoniae* | Amoxicillin | *oqxB_1 aph(3'')-Ia_5*  *blaTEM-168_1*  *fosA_3*  *tet(W)_4*  *blaSHV-108_1* |
| S35 | *A. baumannii* | Co-amoxixlav  Cefuroxime  Piperacillin/Tazocin  Cefpodoxime  Cefoxitin | No genes |
| S36 | *S. aureus* | Penicillin, Erythromycin Ciprofloxacin Trimethoprim | *ermC_13*  *ermC_1*  *ermC_10*  *ermC_2*  *ermC_12*  *dfrG_1* |
| S37 | *P. mirabilis* | Sensitive | *aac(3)-Iid_1*  *blaOXA-1_1*  *dfrA17_1*  *aadA5_1* |
|  | *M morganni* | Gentamicin  Cephalosporin  Septrin  Ciprofloxacin  Trimethoprin  Fosfomycin | *sul1_2*  *aac(3)-lld_1*  *blaOXA-1_1*  *dfrA17_1*  *aadA5_1*  *blaDHA-1_1* |
| S39 | *C. koseri* | Amoxicillin | *BlaCKO-1_1*  *blaMAL-1_2* |
| S42 | *B. cenocepacia* | Gentamicin  Amikacin | ND |
| S44 | *S. marcesens* | Amoxicillin  Co-amoxiclav  Cefuroxime  Cotrimoxazole  Piperacillin/Tazobactam  Ceftazidime | *aac(6')-Ic_1*  *blaSRT-1_1* |
|  | *K. aerogenes* | Amoxicillin  Co-amoxiclav  Cefuroxime  Piperacillin/Tazobactam  Ceftazidime | ND |
| S45 | *C. striatum* | ND | ND |
| S46 | *C. koseri* | Amoxicillin | ND |
| S49 | *K. pneumoniae* | Ciprofloxacin  ESBL | *blaTEM-168_1*  *blaTEM-176_1* |
| S51 | *S. aureus* | Penicillin, Erythromycin | *ermC_13 ermT_2*  *fusB_1*  *blaTEM-171_1* |
|  | *C. koseri* | Amoxicillin | *blaCKO-1_1* |
| S52 | *K. aerogenes* | Penicillin  Cephalosporin | *aac(6')-Ib_1* |
| S54 | *C. striatum* | Penicillin  Ciprofloxacin  Clindamycin | ND |
| S56 | *P. mirabilis* | Amoxicillin  Septrin | *aac(6')-aph(2'')_1*  *blaTEM-1B_1*  *tet(J)_2*  *erm(B)_18* |
|  | *A. fumigatus* | ND | ND |
| S59 | *K. pneumoniae* | Penicillin  Cephalosporin  Septrin  Ciprofloxacin | *aph(6)-Id_1*  *oqxB_1*  *aph(3'')-Ib_5*  *blaOXA-1_1*  *dfrA14_1*  *oqxA_1*  *fosA6_1*  *sul2_2*  *blaTEM-1B_1*  *blaSHV-28_1*  *sul2_15*  *blaSHV-106_1*  *sul1_2*  *tet(A)_4*  *aac(6')-Ib_1*  *fosA_3*  *blaTEM-122_1*  *blaCTX-M-106_1* |
|  | *C. striatum* | Ciprofloxacin  Penicillin  Tetracycline  Clindamycin  Doxycycline | ND |
| S61 | *P. mirabilis* | Sensitive | No genes |
|  | *K. pneumoniae* | Amoxicillin | oqxA  oqxB |
| S62 | *K. aerogenes* | Amoxicillin  Co-Amoxiclav  Cefuroxime  Piperacillin/Tazobatam  Ceftazidime  Cefpodoxime | ND |
|  | *C. striatum* | Ciprofloxacin  Penicillin  Tetracycline  Clindamycin  Doxycycline | ND |
| S63 | *K. pneumoniae* | Co-amoxiclav  Tazocin | sul1_2  aac(6')-lb  fosA6  aph(6)-ld  blaSHV-27 |
|  | *C. striatum* | ND | ND |

^a^Intrinsic and acquired phenotypic resistance reported by culture, ND=Not Done

| **Table S6.** Microbiology, PCR and clinical metagenomics results for all samples processed in this study for the identification of *Aspergillus fumigatus* | | | | | | |
| --- | --- | --- | --- | --- | --- | --- |
| **Patient** | **Respiratory samples** | | | | **Galactomannan** (Positive/Tested) | |
|  |  |  |  |  |  |  |
|  | **Sample Number** | **Organism identified by metagenomic sequencing** | ***Aspergillus fumigatus* qPCR assay (Cq)** | ***Aspergillus* Respiratory Culture** (Positive/Tested) | **BAL > 1.0** | **Serum > 0.5** |
| 26 | S35 | Negative | >40 | Negative |  |  |
|  | Other | Not done | Not done | 0 / 1 | 0 / 1 | 0 / 1 |
| 100 | S39 | Negative | >40 | Negative |  |  |
|  | Other | Not done | Not done | 0 / 5 | 0 / 1 | 0 / 0 |
| 121 | S37 | Negative | >40 | Negative |  |  |
|  | Other | Not done | Not done | 0 / 5 | 0 / 0 | 0 / 0 |
| 177 | S36 | Negative | >40 | Negative |  |  |
|  | Other | Not done | Not done | 0 / 4 | 0 / 0 | 0 / 1 |
| 196 | S42 | Negative | >40 | Negative |  |  |
|  | Other | Not done | Not done | 0 / 2 | 0 / 0 | 0 / 0 |
| 400 | S49 | Negative | >40 | Negative |  |  |
|  | Other | Not done | Not done | 0 / 3 | 0 / 0 | 0 / 1 |
| 408 | S21 | Negative | >40 | Negative |  |  |
|  | Other | Not done | Not done | 0 / 3 | 0 / 0 | 0 / 0 |
| 441 | S51 | Negative | >40 | Negative |  |  |
|  | S20 | Negative | >40 | Negative |  |  |
|  | Other | Not done | Not done | 0 / 4 | 0 / 0 | 0 / 1 |
| 550 | S10 | Negative | >40 | Negative |  |  |
|  | Other | Not done | Not done | 0 / 7 | 0 / 0 | 0 / 2 |
| 563 | S28 | Positive | 31 | Positive |  |  |
|  | Other | Not done | Not done | 3 / 6 | 0 / 1 | 0 / 0 |
| 613 | S18 | Negative | >40 | Negative |  |  |
|  | Other | Not done | Not done | 2 / 2 | 1 / 1 | 0 / 0 |
| 618 | S45 | Negative | >40 | Negative |  |  |
|  | Other | Not done | Not done | 0 / 8 | 0 / 0 | 0 / 0 |
| 677 | S63 | Negative | >40 | Negative |  |  |
|  | S54 | Negative | >40 | Negative |  |  |
|  | S52 | Negative | >40 | Negative |  |  |
|  | Other | Not done | Not done | 0 / 8 | 2 / 2 | 0 / 5 |
| 727 | S53 | Negative | >40 | Negative |  |  |
|  | Other | Not done | Not done | 0 / 0 | 0 / 0 | 0 / 0 |
| 740 | S59 | Negative | >40 | Negative |  |  |
|  | S30 | Negative | >40 | Negative |  |  |
|  | Other | Not done | Not done | 0 / 16 | 1 / 4 | 1 / 2 |
| 749 | S62 | Negative | >40 | Negative |  |  |
|  | S40 | Negative | >40 | Negative |  |  |
|  | Other | Not done | Not done | 0 / 8 | 0 / 1 | 0 / 1 |
| 815 | S46 | Negative | >40 | Negative |  |  |
|  | S25 | Negative | >40 | Negative |  |  |
|  | Other | Not done | Not done | 0 / 5 | 0 / 2 | 0 / 2 |
| 855 | S41 | Negative | >40 | Negative |  |  |
|  | Other | Not done | Not done | 0 / 6 | 0 / 0 | 0 / 0 |
| 872 | S61 | Negative | >40 | Negative |  |  |
|  | S11 | Negative | >40 | Negative |  |  |
|  | Other | Not done | Not done | 0 / 4 | 0 / 1 | 0 / 3 |
| 1033 | S8 | Positive | 33 | Positive |  |  |
|  | Other | Not done | Not done | 0 / 0 | 1 / 1 | 1 / 1 |
| 1036 | S5 | Negative | >40 | Negative |  |  |
|  | Other | Not done | Not done | 0 / 2 | 0 / 0 | 0 / 1 |
| 1054 | S31 | Negative | >40 | Negative |  |  |
|  | Other | Not done | Not done | 0 / 3 | 0 / 0 | 0 / 0 |
| 1065 | S19 | Negative | >40 | Negative |  |  |
|  | S16 | Negative | >40 | Negative |  |  |
|  | Other | Not done | Not done | 0 / 3 | 0 / 1 | 0 / 0 |
| 1069 | S17 | Negative | >40 | Negative |  |  |
|  | Other | Not done | Not done | 0 / 3 | 0 / 0 | 0 / 1 |
| 1082 | S14 | Negative | >40 | Negative |  |  |
|  | Other | Not done | Not done | 0 / 1 | 0 / 0 | 0 / 0 |
| 1092 | S27 | Negative | >40 | Negative |  |  |
|  | Other | Not done | Not done | 0 / 5 | 0 / 2 | 0 / 2 |
| 1262 | S29 | Negative | >40 | Negative |  |  |
|  | Other | Not done | Not done | 0 / 3 | 0 / 1 | 0 / 1 |
| 1292 | S44 | Negative | >40 | Negative |  |  |
|  | Other | Not done | Not done | 0 / 5 | 0 / 3 | 0 / 1 |
| 1346 | S56 | Positive | 32 | Positive |  |  |
|  | Other | Not done | Not done | 0 / 3 | 1 / 1 | 0 / 2 |
| 1440 | S33 | Negative | >40 | Negative |  |  |
|  | Other | Not done | Not done | 0 / 4 | 1 / 2 | 0 / 1 |
| 1457 | S65 | Negative | >40 | Negative |  |  |
|  | S64 | Negative | >40 | Negative |  |  |
|  | Other | Not done | Not done | 0 / 9 | 2 / 2 | 0 / 2 |
| 1503 | S1 | Negative | >40 | Negative |  |  |
|  | Other | Not done | Not done | 0 / 5 | 0 / 0 | 0 / 1 |
| 1512 | S34 | Negative | >40 | Negative |  |  |
|  | Other | Not done | Not done | 0 / 3 | 0 / 1 | 0 / 2 |
| 1583 | S55 | Positive | 31 | Negative |  |  |
|  | Other | Not done | Not done | 4 / 5 | 0 / 0 | 1 / 1 |

**Table S7.** *Klebsiella pneumoniae* and *Corynebacterium striatum* alignment for outbreak analysis**.** (A) The 7 predicted gene multi-locus sequence types for each sample against the *K. pneumoniae* database (numbers in each gene column refer to the allele - a ~ indicates a full length allele similar to the given allele but less than 100% identity)*.* (B) Number of SNPs between each sample of *K. pneumoniae,* (C) number of SNPs analysis in between the two identical CMg samples and two epidemiologically linked *K. pneumoniae* isolates and (D) Number of SNPs between each sample of *Corynebacterium striatum*.

**A**

| **Patient ID** | **Sample** | **ST** | ***gapA*** | ***infB*** | ***mdh*** | ***pgi*** | ***phoE*** | ***rpoB*** | ***tonB*** |
| --- | --- | --- | --- | --- | --- | --- | --- | --- | --- |
| **550** | **S10** | - | 9 | ~4 | ~2 | 1 | 1 | 1 | ~27 |
| **872** | **S11** | 187 | 23 | 31 | 2 | 1 | 9 | 4 | 23 |
| **1054** | **S31** | - | ~4 | 1 | 2 | 52 | 1 | 1 | 7 |
| **1512** | **S34** | 33 | 2 | 3 | 5 | 1 | 12 | 4 | 9 |
| **400** | **S49** | - |  |  |  |  |  |  |  |
| **740** | **S59** | 307 | 4 | 1 | 2 | 52 | 1 | 1 | 7 |
| **872** | **S61** | - |  |  |  |  |  |  |  |
| **677** | **S63** | 661 | 4 | 3 | 1 | 36 | 9 | 10 | 14 |

| **Patient ID** | **Sample** | **S10** | **S11** | **S31** | **S34** | **S59** | **S63** | **S61** |
| --- | --- | --- | --- | --- | --- | --- | --- | --- |
| **550** | **S10** | 0 | 11273 | 11158 | 11322 | 11161 | 12162 | 26023 |
| **872** | **S11** | 11273 | 0 | 11216 | 11512 | 11219 | 11889 | 26148 |
| **1054** | **S31** | 11158 | 11216 | 0 | 11255 | 3 | 12081 | 25874 |
| **1512** | **S34** | 11322 | 11512 | 11255 | 0 | 11258 | 12525 | 25997 |
| **740** | **S59** | 11161 | 11219 | 3 | 11258 | 0 | 12084 | 25877 |
| **677** | **S63** | 12162 | 11889 | 12081 | 12525 | 12084 | 0 | 26929 |
| **872** | **S61** | 26023 | 26148 | 25874 | 25997 | 25877 | 26929 | 0 |

**B**

| **Patient ID** | **301** | **968** | **1054** | **740** | |
| --- | --- | --- | --- | --- | --- |
| **Sample ID** | **KP1** | **KP2** | **S31** | **S59** |  |
| **KP2** | 0 | 5 | 6 | 12 |  |
| **KP1** | 5 | 0 | 14 | 24 |  |
| **S31** | 6 | 14 | 0 | 55 |  |
| **S59** | 12 | 24 | 55 | 0 |  |

**C**

| **Patient ID** | **Sample** | **S45** | **S54** | **S59** | **S52** | **S63** |
| --- | --- | --- | --- | --- | --- | --- |
| **618** | **S45** | 0 | 61 | 115 | 92 | 102 |
| **677** | **S54** | 61 | 0 | 122 | 89 | 97 |
| **740** | **S59** | 115 | 122 | 0 | 157 | 157 |
|  | **S52** | 92 | 89 | 157 | 0 | 30 |
|  | **S63** | 102 | 97 | 157 | 30 | 0 |

**D**

**Table S8. Performance reported after testing different parameters on training set for pathogen identification.** The number of True Positive (TP), False Positive (FP), True Negative and False Negative (TN) samples as well as sensitivity, specificity along and calculated Youden’s Index ((sensitivity+specificity)-1) are presented. (A) Performance reported for a centrifuge score ≥ 338 with different percentages of microbial classified reads. (B) Performance reported for a centrifuge score ≥ 1023 with different percentages of microbial classified reads. (C) Performance reported for a centrifuge score ≥ 2504 with different percentages of microbial classified reads. (D) Performance reported for a centrifuge score ≥ 5838 with different percentages of microbial classified reads. (E) Performance reported for a centrifuge score ≥ 13420 with different percentages of microbial classified reads.

**A**

| **Thresholds** | **0.1%** | **1.0%** | **2.0%** | **5.0%** | **10.0%** | **15.0%** |
| --- | --- | --- | --- | --- | --- | --- |
| **TP** | 35 | 35 | 32 | 31 | 31 | 26 |
| **FP** | 5 | 4 | 2 | 1 | 1 | 1 |
| **TN** | 1 | 2 | 4 | 5 | 5 | 5 |
| **FN** | 0 | 0 | 3 | 4 | 4 | 8 |
| **Sensitivity** | 100% | 100% | 91% | 89% | 89% | 76% |
| **Specificity** | 17% | 33% | 67% | 83% | 83% | 83% |
| **Youden’s Index** | 17% | 33% | 58% | 72% | 72% | 60% |

**B**

| **Thresholds** | **0.1%** | **1.0%** | **2.0%** | **5.0%** | **10.0%** | **15.0%** |
| --- | --- | --- | --- | --- | --- | --- |
| **TP** | 35 | 35 | 33 | 31 | 31 | 28 |
| **FP** | 4 | 4 | 2 | 1 | 1 | 1 |
| **TN** | 2 | 2 | 4 | 5 | 5 | 5 |
| **FN** | 0 | 0 | 2 | 4 | 4 | 7 |
| **Sensitivity** | 100% | 100% | 94% | 89% | 89% | 80% |
| **Specificity** | 33% | 33% | 67% | 80% | 83% | 83% |
| **Youden’s Index** | 33% | 33% | 61% | 72% | 72% | 63% |

**C**

| **Thresholds** | **0.1%** | **1.0%** | **2.0%** | **5.0%** | **10.0%** | **15.0%** |
| --- | --- | --- | --- | --- | --- | --- |
| **TP** | 35 | 35 | 32 | 31 | 30 | 27 |
| **FP** | 4 | 2 | 1 | 1 | 1 | 1 |
| **TN** | 2 | 4 | 5 | 5 | 5 | 5 |
| **FN** | 0 | 0 | 3 | 4 | 5 | 8 |
| **Sensitivity** | 100% | 100% | 91% | 89% | 86% | 77% |
| **Specificity** | 33% | 67% | 83% | 83% | 83% | 83% |
| **Youden’s Index** | 33% | 67% | 75% | 72% | 69% | 60% |

**D**

| **Thresholds** | **0.1%** | **1.0%** | **2.0%** | **5.0%** | **10.0%** | **15.0%** |
| --- | --- | --- | --- | --- | --- | --- |
| **TP** | 35 | 32 | 31 | 31 | 27 | 21 |
| **FP** | 4 | 1 | 1 | 1 | 1 | 1 |
| **TN** | 2 | 5 | 5 | 5 | 5 | 5 |
| **FN** | 0 | 3 | 4 | 4 | 8 | 14 |
| **Sensitivity** | 100% | 91% | 89% | 89% | 77% | 60% |
| **Specificity** | 33% | 83% | 83% | 83% | 83% | 83% |
| **Youden’s Index** | 33% | 75% | 72% | 72% | 60% | 43% |

**E**

| **Thresholds** | **0.1%** | **1.0%** | **2.0%** | **5.0%** | **10.0%** | **15.0%** |
| --- | --- | --- | --- | --- | --- | --- |
| **TP** | 33 | 30 | 28 | 23 | 19 | 15 |
| **FP** | 1 | 1 | 1 | 1 | 1 | 1 |
| **TN** | 5 | 4 | 4 | 4 | 4 | 4 |
| **FN** | 2 | 6 | 7 | 13 | 17 | 21 |
| **Sensitivity** | 94% | 83% | 80% | 64% | 53% | 42% |
| **Specificity** | 83% | 80% | 80% | 80% | 80% | 80% |
| **Youden’s Index** | 78% | 63% | 60% | 44% | 33% | 22% |

**References**

1. Charalampous T, Kay GL, Richardson H, Aydin A, Baldan R, Jeanes C, et al. Nanopore metagenomics enables rapid clinical diagnosis of bacterial lower respiratory infection. Nature biotechnology. 2019;37(7):783-92.

2. Gadsby NJ, McHugh MP, Forbes C, MacKenzie L, Hamilton SKD, Griffith DM, et al. Comparison of Unyvero P55 Pneumonia Cartridge, in-house PCR and culture for the identification of respiratory pathogens and antibiotic resistance in bronchoalveolar lavage fluids in the critical care setting. European journal of clinical microbiology & infectious diseases : official publication of the European Society of Clinical Microbiology. 2019;38(6):1171-8.

3. Langelier C, Kalantar KL, Moazed F, Wilson MR, Crawford ED, Deiss T, et al. Integrating host response and unbiased microbe detection for lower respiratory tract infection diagnosis in critically ill adults. Proceedings of the National Academy of Sciences. 2018;115(52):E12353-E62.

4. Murphy CN, Fowler R, Balada-Llasat JM, Carroll A, Stone H, Akerele O, et al. Multicenter Evaluation of the BioFire FilmArray Pneumonia/Pneumonia Plus Panel for Detection and Quantification of Agents of Lower Respiratory Tract Infection. Journal of clinical microbiology. 2020;58(7):e00128-20.

5. Langelier C, Zinter MS, Kalantar K, Yanik GA, Christenson S, O’Donovan B, et al. Metagenomic Sequencing Detects Respiratory Pathogens in Hematopoietic Cellular Transplant Patients. American Journal of Respiratory and Critical Care Medicine. 2018;197(4):524-8.
