# Supplementary material for: Evaluating the potential for respiratory metagenomics to improve treatment of secondary infection and detection of nosocomial transmission on expanded COVID-19 intensive care units
Source: Genome Med. 2021 Nov 17;13:182. doi: 10.1186/s13073-021-00991-y (PMC8594956; doi:10.1186/s13073-021-00991-y)
Supplement: Supplementary file 2 — Additional file 2: Figs. S1-S3. Fig. S1A-B. Post mortem histological analysis of focal invasive pulmonary aspergillosis (IPA). Fig. S2. Receiver Operator Curve (ROC) curve analysis based on discordant testing (CMg+qPCR) performed for the training set. Fig. S3. WIMP alignment q-score plotted against the equivalent centrifuge score. Tested WIMP alignment q-scores are plotted on the y axis against the equivalent centrifuge score on the x axis. [file 13073_2021_991_MOESM2_ESM.docx]

**Additional File 2: Figures S1-S3.**

**
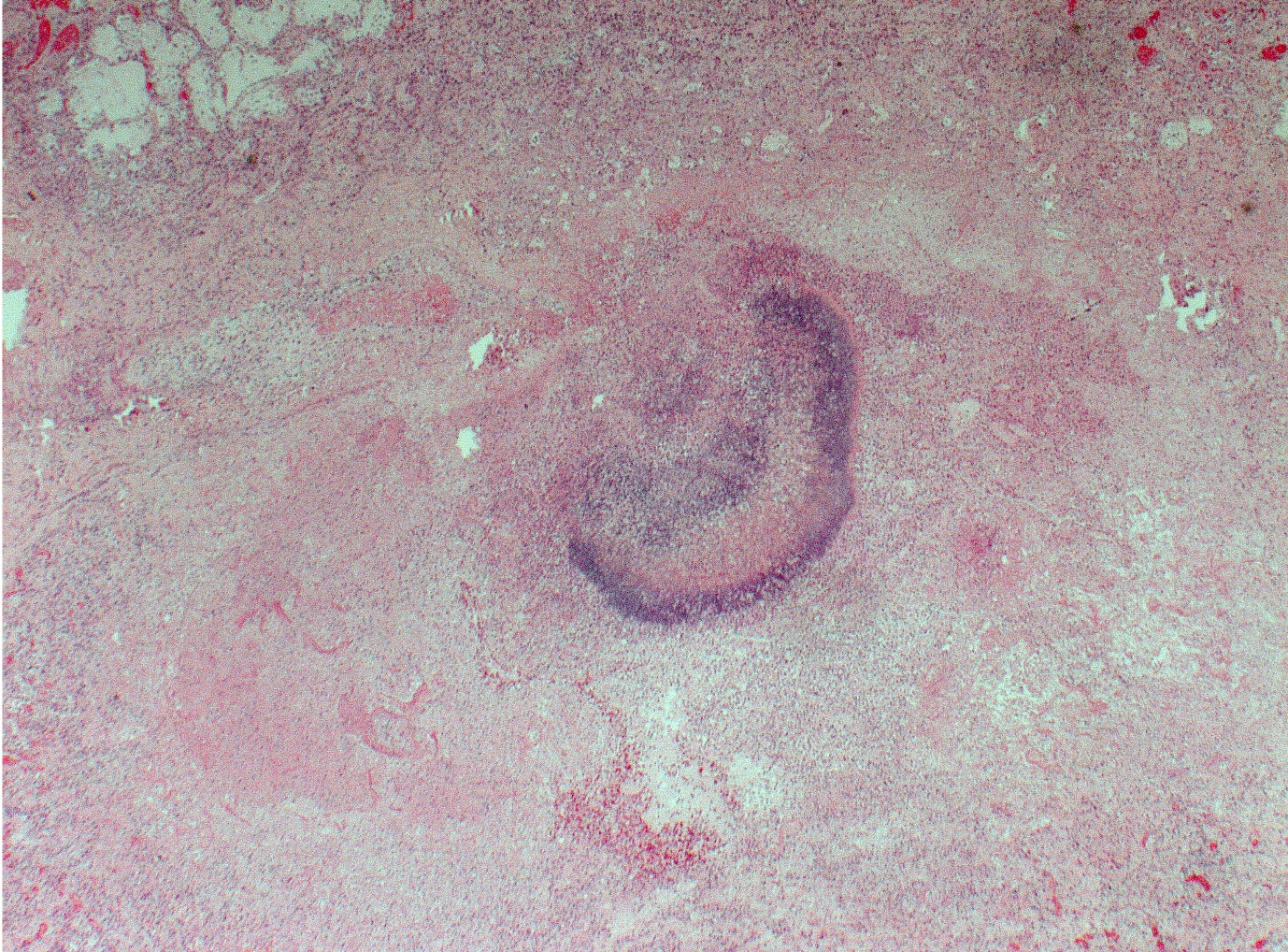
**

**Figure S1A. Post mortem histological analysis of focal invasive pulmonary aspergillosis (IPA)**. Focus of invasive aspergillosis in the left lower lobe involving a large vessel with surrounding lung showing diffuse alveolar damage (approximately 1cm x 1cm) (Haematoxylin & Eosin x 12). This was a single focus identified from ten 3 x 2cm sections of lung.


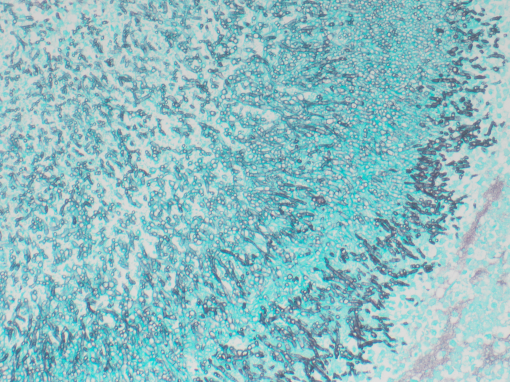


**Figure S1B. Post mortem histological analysis of focal invasive pulmonary aspergillosis (IPA)**. Focal invasive aspergillosis was identified microscopically at post-mortem (Grocott silver stain x 100).


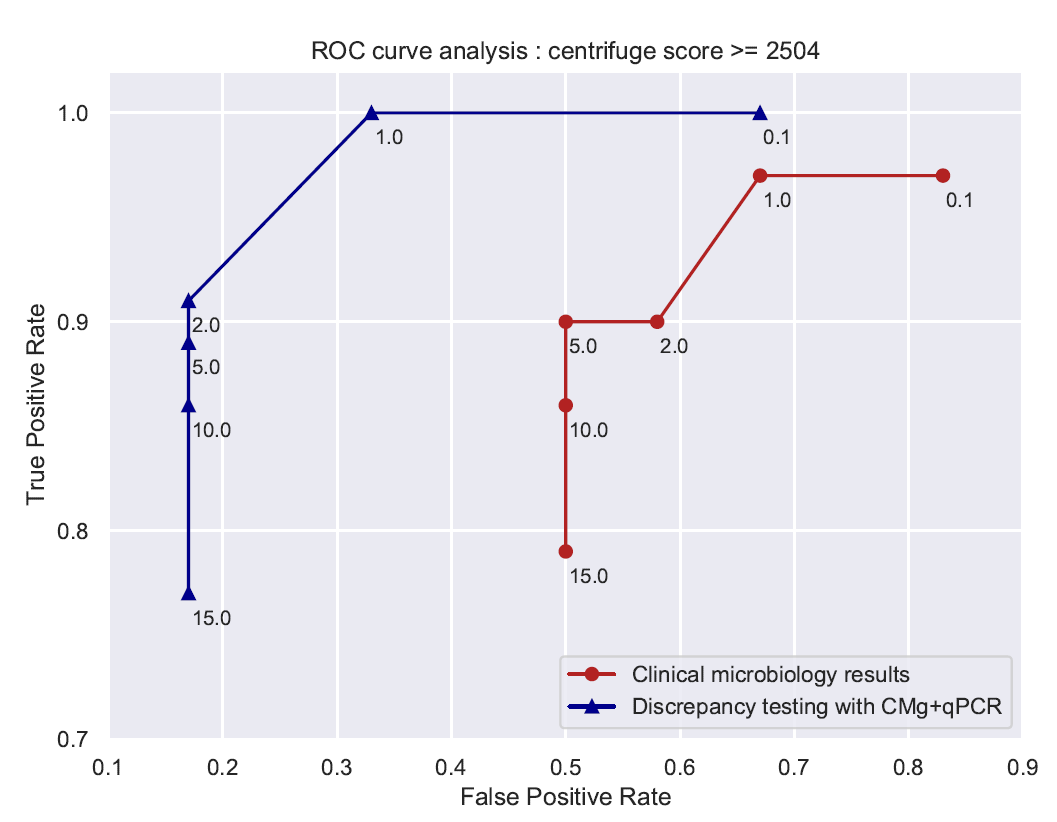


**Figure S2. Receiver Operator Curve (ROC) curve analysis based on discordant testing (CMg+qPCR) performed for the training set.** ROC analysis reported with a centrifuge score ≥2504 tested with different percentages of microbial classified reads. True positive rate (calculated sensitivity) is plotted on the y axis against the false positive rate (1-sensitivity) is plotted on the x axis.


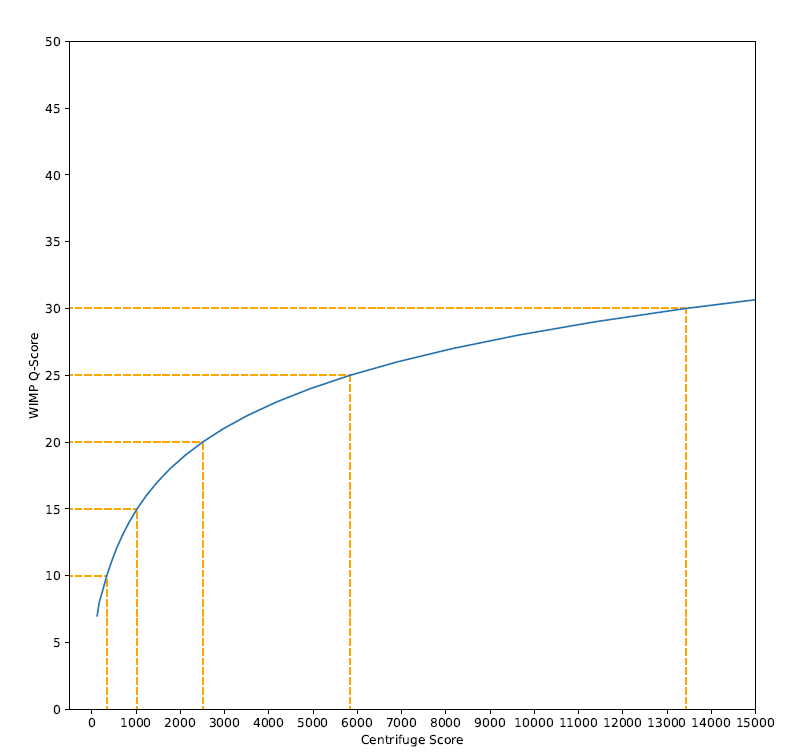


**Figure S3. WIMP alignment q-score plotted against the equivalent centrifuge score.** Tested WIMP alignment q-scores are plotted on the y axis against the equivalent centrifuge score on the x axis.
